# Carbon and Nutrients from Organic Residues Modulate the Dynamics of Prokaryotic and Fungal Communities

**DOI:** 10.3390/microorganisms11122905

**Published:** 2023-12-01

**Authors:** Késia Silva Lourenço, Heitor Cantarella, Eiko Eurya Kuramae

**Affiliations:** 1 Microbial Ecology Department, Netherlands Institute of Ecology (NIOO), Droevendaalsesteeg 10, 6708 PB Wageningen, The Netherlands; lourencokesia@gmail.com; 2 Soils and Environmental Resources Center, Agronomic Institute of Campinas (IAC), Av. Barão de Itapura 1481, Campinas 13020-902, SP, Brazil; cantarella@iac.sp.gov.br; 3 Ecology and Biodiversity, Institute of Environmental Biology, Utrecht University, Padualaan 8, 3584 CH Utrecht, The Netherlands

**Keywords:** organic residue, vinasse, environmental filtering, bacterial community, fungal community, microbial ecology

## Abstract

Inputs of carbon (C) and nutrients from organic residues may select specific microbes and shape the soil microbial community. However, little is known about the abiotic filtering of the same residues with different nutrient concentrations applied to the soil. In our study, we explored how applying organic residue, vinasse, as fertilizer in its natural state (V) versus its concentrated form (CV) impacts soil microbiota. We conducted two field experiments, evaluating soil prokaryotic and fungal communities over 24 and 45 days with vinasse (V or CV) plus N fertilizer. We used 16S rRNA gene and ITS amplicon sequencing. Inorganic N had no significant impact on bacterial and fungal diversity compared to the control. However, the varying concentrations of organic C and nutrients in vinasse significantly influenced the soil microbiome structure, with smaller effects observed for V compared to CV. Prokaryotic and fungal communities were not correlated (co-inertia: RV coefficient = 0.1517, *p* = 0.9708). Vinasse did not change the total bacterial but increased the total fungal abundance. A higher C input enhanced the prokaryotic but reduced the fungal diversity. Our findings highlight vinasse’s role as an abiotic filter shaping soil microbial communities, with distinct effects on prokaryotic and fungal communities. Vinasse primarily selects fast-growing microorganisms, shedding light on the intricate dynamics between organic residues, nutrient concentrations, and soil microbes.

## 1. Introduction

Changes in environmental conditions and resource availability can impact soil biotic interactions both positively and negatively. An example of such a change is the agricultural application of organic residues as fertilizer. When appropriately managed, residue application can increase soil biodiversity, soil quality, plant productivity, and plant health [1,2,3,4,5,6]. All these benefits are linked to the enhancement in soil structure, increased nutrient content, improved water retention, and promotion of microbial activity. Specifically, the macro- and micronutrients in the residues act as abiotic filters by selecting specific microbes and shaping the soil microbial community composition, structure, and function [7,8,9]. In addition, organic residues can also be a source of exogenous microbes (i.e., biotic environmental filters) [1,10,11]. 

The soil microbial community comprises a variety of microbes, including prokaryotes (e.g., bacteria and archaea) and eukaryotes (e.g., fungi and protists), each with its own characteristics [12,13]. Based on niche differentiation, the bacterial and fungal communities are expected to respond differently to the same resource, and these differences may be even greater when the resource differs in composition (C and nutrient concentration), type (organic and inorganic), and form (solid or liquid). Consequently, the co-variation between the two communities will also be different. Lourenço et al. [9] and Lourenço et al. [1] demonstrated that the application of vinasse changed the soil bacterial and fungal community. However, the bacterial community showed resilience, while the fungal community did not, with no correlation observed between communities. Varying the nutrient concentrations within the same type of residue (i.e., the same organic fertilizer) may lead to a gradient of changes in the bacterial and fungal communities. These changes will perhaps be more similar, with stronger co-variation, in the communities that received C than in the communities assembled in the presence of N inorganic fertilizer (different types and forms of fertilizer) [14,15]. 

In this study, we investigated how alterations in the carbon and nutrient concentrations of an organic residue, used as fertilizer, impact the soil microbial communities. Increases in C and nutrients are expected to increase the abundance and activity of fast-growing microbes [16]. To achieve our objective, we used a sugarcane field, which is an ideal model agricultural system for our purposes because large amounts of organic residues, such as harvest straw (approximately 8–15 t ha^−1^ of dry matter), are left on the soil. Additionally, vinasse is commonly applied as organic fertilizer (rates of 50 to 150 m^3^ ha^−1^ per season) [17]. Vinasse is a residue of sugarcane ethanol production. In Brazil, approximately 381 billion liters of vinasse are produced each year and recycled in sugarcane fields [18]. Applying this residue to the soil as fertilizer is challenging because vinasse is a liquid with a low total solid content of 25.15 g L^−1^ [17]. To reduce the cost of applying vinasse far from the sugar mill, vinasse is frequently concentrated through evaporation, which increases the concentrations of organic C and nutrients. A diverse range of C and nutrient loads are applied to the soil depending on whether “in nature” (V) or concentrated vinasse (CV) is used, which may alter the impact of these organic amendments on the soil microbial community. Application of mineral fertilizers is a standard practice in sugarcane fields, so it is always applied, with or without vinasse. However, it is not known how changes in C and nutrient concentration from vinasse can impact the structure of soil prokaryote and fungal communities in dry and rainy seasons. Given this context, our goal was to evaluate how differences in C and macro- and micronutrients from vinasse can shape the soil microbiome using common management strategies adopted by sugarcane mills. 

## 2. Materials and Methods

### 2.1. Experimental Setup

This study comprised a subset of treatments of two former field experiments [19] with sugarcane in which vinasse, i.e., a liquid residue from ethanol production, was recycled. The experimental site is situated in Piracicaba, Brazil, at the Paulista Agency for Agribusiness Technology (APTA) (22°41′ S; 47°33′ W). The local climate is classified as humid tropical [20]. The first experiment was carried out during the rainy season (RS), spanning from 13 December 2013 to October 2014, while the second experiment was carried out in the dry season (DS), from 15 August 2014 to August 2015. The experiments were located side-by-side within the same experimental field and consequently had similar soil, Oxisol/Ferrasol [21,22]. The soil properties (physical and chemical) [23,24] for the 0 to 20 cm soil layer are shown in Table 1. The sugarcane fields were previously mechanically harvested without burning the crop (green cane), in accordance with current legislation, and 12 and 16 Mg ha^−1^ of straw was present on top of the soil in RS and DS, respectively. Daily temperature and precipitation measurements throughout the experiments were obtained from a meteorological station located near the experimental area (Figure S1). 

The following treatments were applied. Control: plots without fertilization; N: 100 kg N ha^−1^ of NH_4_NO_3_ (ammonium nitrate); V + N: vinasse plus NH_4_NO_3_; and CV + N: concentrated vinasse plus NH_4_NO_3_. The experiments were conducted in a randomized block design with three replicates. In all treatments, inorganic N fertilizer was applied at 100 kg N ha^−1^ as NH_4_NO_3_, the most common fertilizer for green cane. The application of N fertilizer and both vinasses followed common practices in the sugarcane industry in the state of Sao Paulo, Brazil. Inorganic N was surface-applied close to the plant row (0.1 m) covering approximately 20% of the sugarcane field (2000 m^2^ ha^−1^). V was sprayed at a rate of 100 m^3^ ha^−1^ (10 L m^−2^) over the entire experimental plot (10,000 m^2^ ha^−1^). By contrast, due to its high nutrient concentration, CV was surface-applied at a rate of 17 m^3^ ha^−1^ (8.5 L m^−2^) close to the plant row (0.1 m), similar to the inorganic N fertilizer, rather than broadcast over the whole field; approximately 20% of the sugarcane field received CV (2000 m^2^ ha^−1^). The CV rate was based on the average level of concentration of vinasse by sugar mills (5.8 times) (Table 2). 

### 2.2. Soil Sampling and DNA Extraction

Six samples of soil were collected from the 0 to 10 cm layer of each plot at three time points in each experiment. The soil was sampled in the fertilized area, specifically in close proximity to the sugarcane plants. The soil samples were collected at 7, 22, and 24 days after fertilization in RS (on 20 December 2013, 4 January 2014, and 6 January 2014, respectively) and 11, 19, and 45 days after fertilization in DS (on 26 August 2014, 3 September 2014, and 29 September 2014, respectively). The different time points were selected based on the high CO_2_ and N_2_O emissions observed in the same soils and samples in our previous study [25]. Soil samples (30 g) were stored at −80 °C for molecular analyses. DNA was extracted from 0.30 g of soil using the MoBio PowerSoil DNA Isolation Kit (MoBio, Solana Beach, CA, USA), according to the manufacturer’s instructions. The quality of the DNA was confirmed through separation using electrophoresis on a 1% (*w*/*v*) agarose gel under UV light, and the quantity of DNA was determined using a Qubit 2.0 Fluorometer (Life Technologies, Carlsbad, CA, USA). 

### 2.3. Prokaryotic (16S rRNA) and Fungal (ITS) Amplicon Sequence Processing 

The total DNA was used as the template for amplification of the 16S rRNA gene and internal transcribed spacer (*ITS*) using the primer sets 515FP1/806RP1 and ITS1/ITS2 for prokaryotic and fungi, respectively (Supplementary Table S1). Amplification and sequencing were performed on the Illumina MiSeq System at Genome Québec, Montréal, Canada. Dual-index and Illumina sequencing adapters were attached to the amplicons. After library quantification, normalization, and pooling, MiSeq V3 reagent kits were used to prepare the samples for MiSeq sequencing. 

The raw sequencing data were processed using the Hydra pipeline version 1.3.3 [26] implemented in Snakemake [27]. Adapters and PhiX contaminants were removed using BBDuk2 in the BBMap tool suite [28]. Paired ends were merged using the fastq_mergepairs option from vsearch [29]. The ITS2 region was extracted from ITS sequences using ITSx version 1.011 [30]. Sequence clustering into OTUs was performed using vsearch via the UPARSE strategy through dereplication, sorting by abundance with at least two sequences and clustering using the UCLUST smallmem algorithm [31]. The UCHIME algorithm implemented in VSEARCH was used to detect and remove chimeric sequences in de novo mode [32]. Before the dereplication step, all reads were mapped to OTUs with the usearch_global method implemented in VSEARCH to generate an OTU table and convert it to BIOM-Format [33]. For 16S rRNA gene sequences, taxonomic information for each OTU was added to the BIOM file by aligning the sequences to the SILVA database (release 132) [33] using the SINA classifier [34]. For ITS sequences, taxonomic information was added to the BIOM file by running the RDP Classifier re-trained on the UNITE database release 8.2 [35]. 

### 2.4. Real-Time PCR

The total DNA used for 16S rRNA gene and ITS amplicon sequencing was also used for real-time PCR to quantify the total bacteria and fungi in the different treatments. The abundances of the 16S rRNA (bacteria) and 18S rRNA (fungi) genes were quantified in duplicate through real-time PCR in a BIORAD CFX96 Touch™ Real-Time PCR Detection System. The amplification and thermal cycler conditions are described in Supplementary Table S1. The efficiencies of qPCR for the 16S rRNA and 18S rRNA genes were 96% (R^2^ = 0.99) and 88% (R^2^ = 0.99), respectively. Each qPCR run included a duplicate DNA template, a standard positive control, and a negative control. Plasmid DNA containing fragments of 16S rRNA and 18 rRNA genes were used as standards. The qPCR amplicon products were checked using melting curve analysis and agarose gel electrophoresis.

### 2.5. Statistical Analyses

Rarefaction curves from non-rarefied data, using the sequence sample size and number of different OTUs, were used to indicate whether the measurement depth met the requirements (Figure S2). The 16S rRNA gene and ITS sequence data were rarefied (randomly subsampled) to the size of the smallest sample prior to alpha and beta diversity analyses. The 16S rRNA gene and ITS datasets were rarefied to 23,346 and 18,899 sequences, respectively, for the RS experiment and 18,814 and 12,964 sequences, respectively, for the DS experiment. Estimates of alpha and beta diversity were calculated in RStudio version 1.0.136 running R version 3.3.1 using the phyloseq package [36]. The estimates for alpha diversity included the richness, Chao1, Simpson, and Shannon diversity indices [37]. Differences among treatments were evaluated using ANOVA and Tukey’s test (*p* < 0.05). 

To assess the effects of the treatments on the total bacterial, archaeal, and fungal community compositions, the rarefied Hellinger-transformed data at the family level were ordinated through principal coordinate analysis (PCoA) using the Bray distance measure. Permutational multivariate analysis of variance (PERMANOVA), using the “anosim” command in the “vegan” package, was used to ascertain group significance with 9999 permutations [38]. The tested factors included treatment (control, N, V + N, and CV + N), time (RS: 7, 22 and 24 days and DS: 11, 19, and 45 days), and their interactions. In parallel, the data were ordinated using analysis of similarities (ANOSIM), and group significance was assessed through between-group analysis applying a random permutation test (9999 repetitions) in the “ade4” package [39]. Hellinger data transformation was used to test the effects of the treatments on bacterial and fungal community compositions.

The link between prokaryote and fungal community compositions was assessed using the Procrustes approach expressed in terms of m^2^ [40,41] and tested with 9999 permutations using the Monte Carlo test [42] using the “vegan” package [38] in R software 3.6.3. The higher the value of m^2^, the weaker the relationship between the microbial communities. Finally, co-inertia analysis computed using the “ade4” package in R [39] was applied to the rarefied and Hellinger-transformed data at the family level. Co-inertia analysis is a multivariate method for coupling two tables and is quantitatively defined by an RV coefficient that measures the correlation between two tables (RV values: 0 to 1) [43,44]. RV coefficients close to one indicate strong relationships between the datasets.

Bacterial/archaeal and fungal families that were significantly associated with the treatments were identified using the Random Forest (RF) algorithm executed in the web-based tool Microbiome Analyst [45]. We used 1000 trees; each tree was constructed using a different bootstrap sample from the original data. Ultimately, the out-of-bag (OOB) error estimate is determined based on the proportion of times that the tree is not equal to the true class of the tree averaged over all cases. In addition, the algorithm identifies the main families responsible for the difference.

## 3. Results

### 3.1. Bacterial and Fungal Community Structures

For the 16S rRNA partial gene, an average of 32,870 (20,489–58,087) reads per sample was obtained, with a total of 2,333,773 high-quality sequences clustered into 9573 OTUs. For the fungal community represented by ITS amplicon sequences, an average of 25,013 (13,087–49,104 reads) reads per sample was obtained, with a total of 1,775,918 sequences clustered into 2839 OTUs. The sequencing depth indicated that the diversities of the prokaryotic and fungal soil communities were adequately captured (see Supplementary Figure S2).

The alpha diversity (OTU richness, Chao1, Simpson, and Shannon) of the bacterial and fungal communities was affected by the treatment but not by time (Table S2, Figures S3 and S4). The OTU richness and Chao1 and Simpson indices for the bacterial communities were higher in RS than in DS (Figures S3 and S4). The treatments altered the diversity of both the prokaryotic and fungal communities in both experiments, with no changes by day (*p* ≤ 0.05), except for the bacterial community in RS (Table S2). Similar patterns of increases in the Simpson and Shannon indices of the prokaryotic community for treatments with vinasse were observed in both experiments. By contrast, the diversity indices of the fungal community were dramatically lower in the CV + N and V + N treatments than in the control and inorganic N treatment (Figure S4). 

**Figure 1:**
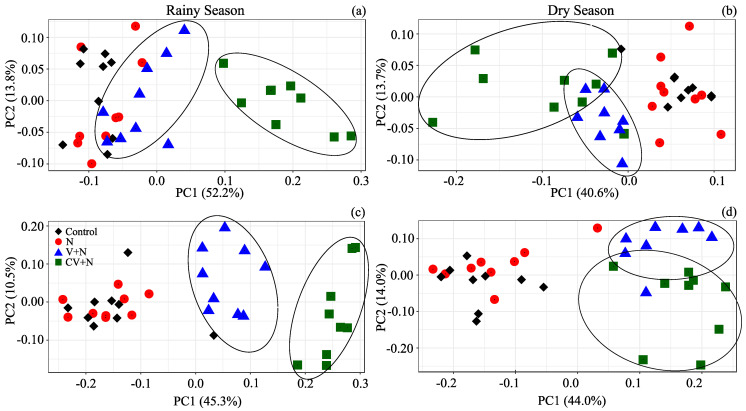
Changes in the soil bacterial plus archaea (**a**,**b**) and total fungal (**c**,**d**) communities in the rainy (**a**,**c**) and dry seasons (**b**,**d**) as depicted by Bray–Curtis dissimilarity. The treatments were as follows: control; N: inorganic fertilizer ammonium nitrate; V + N: non-concentrated vinasse plus ammonium nitrate; and CV + N: concentrated vinasse plus ammonium nitrate. Each point represents an individual sample, with colors indicating different treatments. The positions of the points are the average for the jackknife replicates.

PERMANOVA and PCoA revealed a clear separation among the treatments at the family level (PERMANOVA: *p* = 0.00 for RS and DS). The interaction between treatment and time was not significant (PERMANOVA: *p* = 0.41 and *p* = 0.24 for RS and DS) (Figure 1a,b; Figure S5a,b; Table S3). Differences in fungal community structure were only observed among the treatments in both experiments (PERMANOVA: *p* = 0.00) (Figure 1c,d; Figure S5c,d; Table S3). In summary, CV + N and V + N application increased the bacterial/archaeal diversity and decreased the fungal diversity.

To measure the co-variation between the bacterial/archaeal community and fungal community, we performed Procrustes and co-inertia analyses. A non-significant concordance between ordinations was found (Procrustes: m^2^ = 122.22 based on 9999 permutations; co-inertia: RV coefficient = 0.1517, *p* = 0.9708, based on 9999 replicates), suggesting that the changes in the structure of the bacterial/archaeal community were not associated with changes in the fungal community. 

### 3.2. Differences in Microbial Taxa between Residues

The bacterial and archaeal communities were composed of 29 different phyla. The bacterial community was dominated by Actinobacteria (25.6%), Proteobacteria (23.1%), Acidobacteria (17.3%), Chloroflexi (13.1%), Planctomycetes (6.8%), Gemmatimonadetes (3.8%), Verrucomicrobia (2.7%), Bacteroidetes (2.1%), Firmicutes (1.4%), and Rokubacteria (1.4%), while the archaeal community mainly comprised Thaumarchaeota (0.6%) and Euryarchaeota (0.1%) (Figure S6). The CV + N and V + N treatments altered the microbial community at the phylum level in both experiments, with increases in Actinobacteria, Proteobacteria, Bacteroidetes, and Firmicutes and decreases in Acidobacteria and Chloroflexi compared with the inorganic N treatment and the control (Figures S7 and S8). The fungal community was composed of nine phyla, mainly Ascomycota (42.8%), Basidiomycota (4.5%), Mortierellomycota (0.8%), and Chytridiomycota (0.1%); unclassified and unidentified fungal phyla represented 51.7% of the total community. Compared with the control and inorganic N treatment, the CV + N and V + N treatments exhibited decreases in the abundances of Ascomycota and Basidiomycota and large increases in unclassified and unidentified phyla (Figure S9).

**Figure 2:**
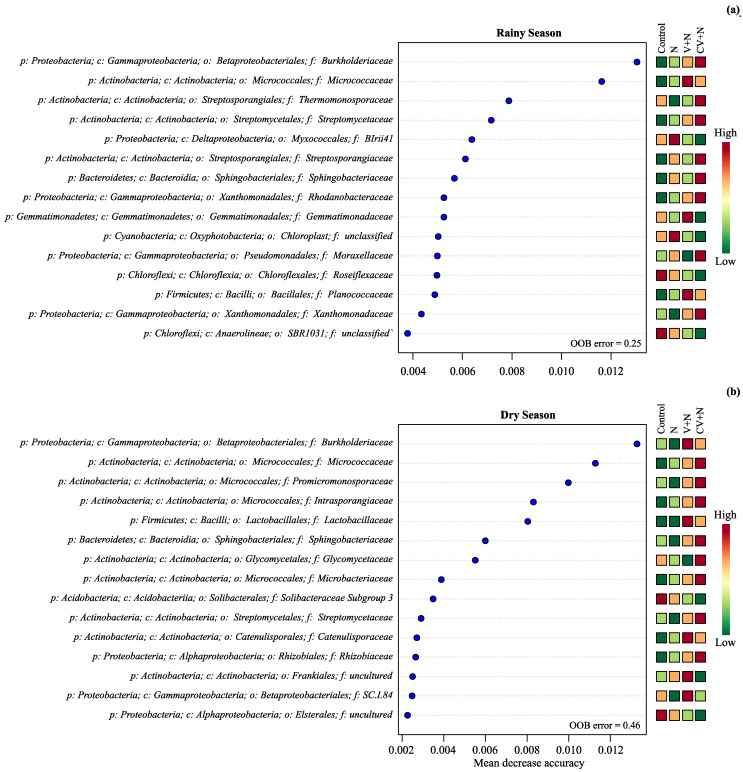
Variable importance for predicting changes in the soil bacterial plus archaea community in the rainy (**a**) and dry season (**b**) in a Random Forest (RF) classification. Higher values of the mean decrease in accuracy indicate variables that are more important to the classification. p:, c:, o:, and f: mean phylum, class, order, and family level, respectively.

The bacterial families responsible for the differences among the treatments in both experiments mainly belonged to the phyla Acidobacteria, Actinobacteria, Bacteroidetes, Chloroflexi, Firmicutes, Gemmatimonadetes, and Proteobacteria (Figure 2a,b). In RS, high proportions of Actinobacteria (*Micrococcaceae*), Firmicutes (*Planococcaceae*), and Gemmatimonadetes (*Gemmatimonadaceae*) were found in the V + N treatment, whereas Actinobacteria (*Streptosporangiaceae*, *Streptomycetaceae*, *Thermomonosporaceae*), Bacteroidetes (*Sphingobacteriaceae*), and Gammaproteobacteria (*Burkholderiaceae*, *Moraxellaceae*, *Rhodanobacteraceae*, *Xanthomonadaceae*) were overrepresented in CV + N treatment (Figure 2a). In the inorganic N treatment, the relative abundance of Chloroflexi (*Roseiflexaceae*), Cyanobacteria (order Chloroplast; family unclassified), and Proteobacteria (order Myxococcales; family *BIrii41*) were higher than in the CV + N and V + N treatments, whereas Chloroflexi (class Anaerolineae; order SBR1031; and family unclassified) was more abundant in the control than in the CV + N and V + N treatments. Similar to the results in RS, the families responsible for the differences among the treatments in DS mainly increased in the CV + N and V + N treatments (Figure 2b). The families that increased most in abundance in the V + N treatment belonged to the phyla Actinobacteria (*Catenulisporaceae*, order Frankiales, family uncultured), Gammaproteobacteria (*Burkholderiaceae*, order Betaproteobacteriales, family *SC.I.84*), and Firmicutes (*Lactobacillaceae*), whereas the phyla Actinobacteria (*Micrococcaceae*, *Promicromonosporaceae*, *Intrasporangiaceae*, *Glycomycetaceae*, *Microbacteriaceae*, *Streptomycetaceae*), Bacteroidetes (*Sphingobacteriaceae*), and Proteobacteria (*Rhizobiaceae*) increased most in abundance in the CV + N treatment. Acidobacteria (*Solibacteraceae Subgroup 3*) and Alphaproteobacteria (order Elsterales family uncultured) were the only taxa that increased in abundance in the control.

**Figure 3:**
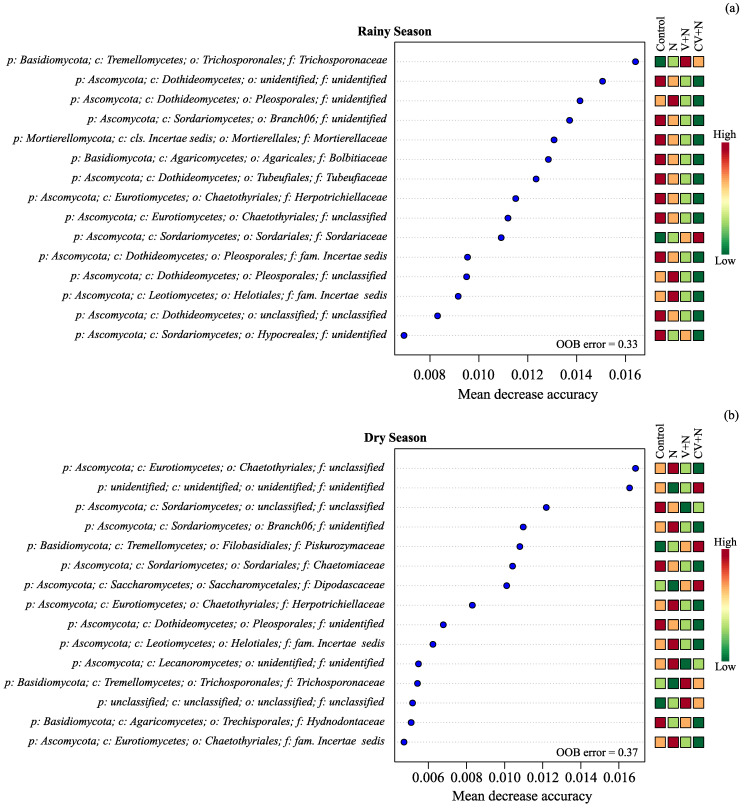
Variable importance for predicting changes in the soil fungal community in the rainy (**a**) and dry season (**b**) in a Random Forest (RF) classification. Higher values of the mean decrease in accuracy indicate variables that are more important to the classification. p:, c:, o:, and f: mean phylum, class, order, and family level, respectively.

The main fungal taxonomic biomarkers responsible for the differences among treatments were more abundant in the control and inorganic N treatments due to the decrease in fungal diversity in the CV + N and V + N treatments (Figure 3a,b). However, the relative abundances of specific orders increased in the treatments with vinasse, such as Trichosporonales (*Trichosporonaceae*) in the V + N treatment and Sordariales (*Sordariaceae*), Filobasidiales (*Piskurozymaceae*), and Saccharomycetales (*Dipodascaceae*) in the CV + N treatment.

### 3.3. Abundances of Total Bacteria and Total Fungi

**Figure 4:**
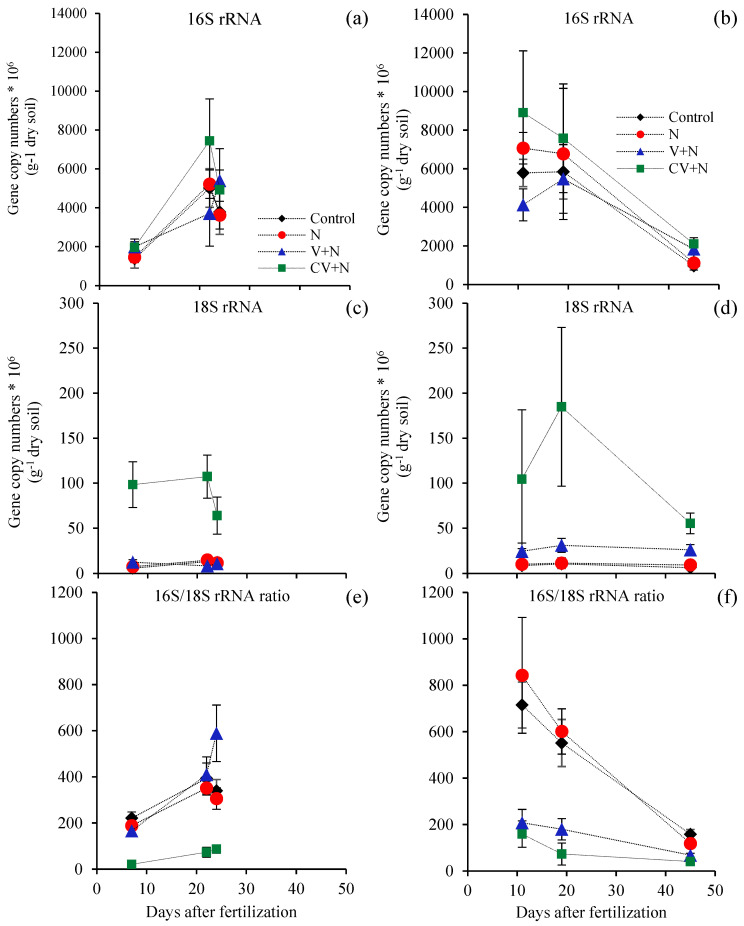
Gene copy numbers per gram of dry soil of total bacteria (16S rRNA) (**a**,**b**) and total fungi (18S rRNA) (**c**,**d**) and 16S/18S rRNA ratio (**e**,**f**) obtained using qPCR from soil with sugarcane in different treatments in (**a**,**c**,**e**) rainy and (**b**,**d**,**f**) dry seasons. The treatments are control; N: mineral N fertilizer, ammonium nitrate; V + N: non-concentrated vinasse plus mineral N; CV + N: concentrated vinasse plus mineral N (n = 3).

The abundances of total bacteria and total fungi, as determined using real-time PCR, are illustrated in Figure 4 and Table S5 for all treatments and sampling time points in both experiments. Vinasse application, regardless of concentration, had no impact on total bacterial abundance. However, the total bacterial abundance changed with the sampling time point. By contrast, CV application increased the total fungal abundance in the soil in both experiments (Table S5), with no difference between time points. The ratio of total bacterial abundance and total fungal abundance (16S/18S rRNA) decreased in the treatments with vinasse, except in the V + N treatment in RS, for which the ratio was similar to that of the control (Figure 4e,f). A low ratio indicates that fungal abundance increased relative to bacterial abundance.

## 4. Discussion

The chemical composition of the vinasse acted as an environmental filter, modulating specific microbes and consequently changing the dynamics of the soil microbial community. Mineral fertilizer alone did not exhibit such filtering capacity, probably due to the lack of C (Suleiman et al., 2018). Vinasse is rich in organic C and N compounds, in addition to potassium, phosphorus, calcium, and micronutrients (Parnaudeau et al., 2008, Mutton et al., 2014), which greatly influence soil microbiome activity as well as N_2_O and CO_2_ production [1,9,19,25,46,47,48]. In the current study, the input of nutrients to the soil via V and CV modulated and increased soil microbial community activity. However, as the nutrient load was higher in CV, applying CV resulted in dramatically larger changes in the soil microbiome than applying V. Hence, the increased availability of organic resources was the main factor responsible for the changes in the soil microbial community.

The addition of the two vinasses with different concentrations of nutrients impacted both the bacterial and fungal communities (Figure 1), but the responses of the two communities differed. Although the diversity of the bacterial community increased, the total bacterial abundance remained the same. By contrast, the application of CV increased the total fungal abundance 11-fold compared with the control treatment but did not increase fungal diversity; on the contrary, fungal alpha diversity decreased when the vinasses were applied to the soil. The different responses of the two communities are related to lifestyle strategies and resource preferences. Studies have shown that when V is applied together with mineral N, the resident soil bacterial community is not resistant but is instead highly resilient, whereas the resident soil fungal community is neither resistant nor resilient [1,9]. Fungi are decomposers with a lower metabolic nutrient demand and wider enzymatic capabilities than bacteria. These characteristics allow them to mineralize low-quality substrates, such as sugarcane straw [49]. The organic C and N and mineral nutrients present in the vinasses promoted the abundance of specific fungi capable of degrading the large amounts of harvest straw present on the soil. Bacteria are also decomposers but are characterized by a faster turnover and higher metabolic activities than fungi [50,51]. Consequently, bacteria act as rapid recyclers of simple C compounds [52]. 

The main bacterial families that benefited from vinasse addition belonged to the phyla Actinobacteria, Proteobacteria, and Firmicutes, such as *Micrococcaceae*, *Streptomycetaceae, Burkhoderiaceae,* and *Lactobacilillaceae*. *Micrococcaceae*, for example, was most abundant in the V + N treatment in RS and the CV + N treatment in DS. These findings corroborate our previous finding that applying V as an organic residue increases *Micrococcaceae* [1]. *Micrococcaceae* [53] and *Streptomycetaceae* [54] are primary decomposers with a well-known cellulolytic activity. These results indicate that organic fertilization affected the abundance of straw-degrading species, probably due to the supply of C. Members of Firmicutes are usually present in vinasse or increase in the soil after vinasse application [55,56]. The most important family for predicting changes in the soil bacterial community was *Burkhoderiaceae*. This family is extremely diverse and includes saprophytic organisms, phytopathogens, opportunistic pathogens, and growth-promoting bacteria. Some *Burkhoderiaceae* members fix nitrogen or release iron or phosphorus from rock phosphates [57,58]. Moreover, *Burkholderia* spp. has reportedly been isolated from sugarcane plants [59,60].

The fungal families that increased the most in the treatments with vinasse were *Trichosporonaceae* and *Sordariaceae* in RS and *Dipodascaceae, Piskurozymaceae,* and *Trichosporonaceae* in DS. Members of *Trichosporonaceae* use organic N (ethylamine, L-lysine, and cadaverine) and nitrite as sources of N and are linked to the decomposition of hemicellulose and the assimilation of phenolic compounds [61,62]. Vinasses contain glycerol, ethanol, lactic and acetic acids, and phenolic compounds [63,64], which likely favor the activities of *Trichosporonaceae* family members. The *Sordariaceae* family is saprobic and feeds on wood, rotting vegetation, and dung in terrestrial habitats. Most species of this family are coprophilous and usually live or grow on dung. The increase in their relative abundance was probably due to the rich environment in the treatments with vinasse. Members of the *Piskurozymaceae* family can use nitrate and assimilate low-molecular-weight aromatic compounds such as D-glucose, D-galactose, D-ribose, D-arabinose, sucrose, lactose, glycerol, L-arabitol, and ethanol, which are all present in vinasse [65,66,67]. Similar to our results, Zhang et al. [68] observed an increase in *Piskurozymaceae* members when organic residues were applied to soil. However, the most important fungal families for predicting changes in the soil fungal community in both experiments were families that were overrepresented in the control and inorganic N treatments, as alpha diversity decreased in the treatments with vinasse.

## 5. Conclusions

Our results indicate that the nutrients present in the two vinasses increased copiotrophic microorganisms, particularly fungi. Vinasse increased bacterial community diversity but decreased fungal community diversity. The presence of straw (high C:N ratio, ~100:1) and the nutrients in the vinasses likely acted as an environmental filter that shifted the community toward microbes related to C compound degradation, especially fungi. However, these changes were smaller when V (lower nutrient load) was applied than when CV was applied. The choice to apply V or CV is driven by economic and logistic factors. Sugar mills, cultivating areas as large as 10–20 thousand hectares, may invest in one type of vinasse or the other but rarely consider the consequences and impact of the vinasse type on the soil microbial community. This, in turn, affects the speed of straw mulch decomposition, biological nitrogen fixation, and pathogen suppression.

## Figures and Tables

**Table 1:** Properties of the soils used.

		Rainy Season (RS)	Dry Season (DS)
Soil Layer	cm	0–20	0–20
Bulk density	g cm^−3^	1.42	1.48
pH ^a^	-	5.3	5.0
OM ^b^	g dm^−3^	23	21
P ^c^	mg dm^−3^	10	15
K	mmol_c_dm^−3^	0.5	0.7
Ca	mmol_c_dm^−3^	45	17
Mg	mmol_c_dm^−3^	20	12
H + Al ^d^	mmol_c_dm^−3^	31	35
CEC ^e^	mmol_c_dm^−3^	98	65
Soil texture ^f^			
Clay	g kg^−1^	619	631
Silt	g kg^−1^	145	151
Sand	g kg^−1^	236	218

^a^ (CaCl_2_; 0.0125 mol L^−1^); ^b^ organic matter; ^c^ available phosphorus, and K, Ca, and Mg were extracted with ion exchange resin; ^d^ buffer solution (pH 7.0); ^e^ cation exchange capacity; ^f^ soil texture determined through the densimeter method.

**Table 2:** Chemical characteristics of the vinasse applied in the experiments.

		Rainy Season—RS ^a^		Dry Season—DS	
		CV ^b^	V	CV	V
pH		4.0	4.1	4.2	3.9
Density	(g mL^−1^)	1.02	0.95	0.99	0.95
Total mineral residue	(g L^−1^)	43.76	5.84	44.21	12.05
C org	(g L^−1^)	69.7	25.7	65.3	31.4
N tot	(g L^−1^)	2.80	0.53	3.0	0.9
NH_4_^+^-N	(mg L^−1^)	119.8	63.4	100.9	41.6
NO_3_^−^-N	(mg L^−1^)	21.2	10.8	23.7	4.1
C/N		25/1	49/1	22/1	35/1
P	(g kg^−1^)	1.00	0.17	0.53	0.23
K	(g kg^−1^)	17.3	2.6	21.0	4.7
Ca	(g L^−1^)	5.49	0.79	5.9	1.41
Mg	(g L^−1^)	1.45	0.02	2.38	0.54
S	(g L^−1^)	2.78	0.40	4.60	0.99
Cu	ppm	3.00	1.00	5.00	2.00
Mn	ppm	48.00	7.00	26.00	6.00
Zn	ppm	7.00	1.00	5.00	3.00
Fe	ppm	249.00	39.00	8.00	2.00

^a^ RS: rainy season (2013/2014 cycle); DS: dry season (2014/2015 cycle), ^b^ CV: concentrated vinasse; V: non-concentrated vinasse.

## Data Availability

The raw sequences were submitted to the European Nucleotide Archive (ENA) under study accession number PRJEB44846.

## Supplementary Materials

The following supporting information can be downloaded at https://www.mdpi.com/article/10.3390/microorganisms11122905/s1. Figure S1. Rainfall (mm) and air temperature (°C) measured in the (a) rainy and (b) dry seasons. Figure S2. Rarefaction curves from non-rarefied data from the bacterial plus archaea (a) and fungi (b) communities in each treatment. The treatments were: Control; N: inorganic fertilizer ammonium nitrate; V+N: non-concentrated vinasse plus ammonium nitrate; and CV+N: concentrated vinasse plus ammonium nitrate. Figure S3. Alpha diversity of the bacterial and archaea families in different treatments in the rainy (a, b, c, d) and dry season (e, f, g, h), without timepoint comparisons. The treatments were: Control; N: inorganic N fertilizer; V+N: non-concentrated vinasse plus inorganic N fertilizer; and CV+N: concentrated vinasse plus inorganic N fertilizer. Means followed by the same letter at each treatment do not differ significantly by the Tukey’s test (Significant difference: *p* ≤ 0.05; ns: Non-significant). Figure S4. Alpha diversity of the total fungal families in different treatments in the rainy and dry season (a, b, c, d), without timepoint comparisons. The treatments were: Control; N: inorganic N fertilizer; V+N: non-concentrated vinasse plus inorganic N fertilizer; and CV+N: concentrated vinasse plus inorganic N fertilizer. Means followed by the same letter at each treatment do not differ significantly by the Tukey’s test (Significant difference: *p* ≤ 0.05; ns: Non-significant). Figure S5. Temporal changes in the soil bacterial and archaea (a, b), and total fungal (c, d) communities in the rainy (a, c) and dry (b, d) season as depicted by Bray-Curtis dissimilarity (which accounts for changes in the relative abundance of families). Principal coordinate analysis (PCoA) of soils cultivated with sugarcane was performed with three time points. To illustrate the differences between days in each treatment, each time point was showed with different colour. The treatments were as follows: Control; N: inorganic fertilizer ammonium nitrate; V+N: non-concentrated vinasse plus ammonium nitrate; and CV+N: concentrated vinasse plus ammonium nitrate. Each point represents an individual sample, with colors indicating days. The positions of the points are the average for the jackknife replicates. OUT table rarefied. Figure S6. Sequence abundance phylum of Bacteria and Archaea Domains (>0.002) using normalized values between 0 and 1 (%) for Control; N: inorganic fertilizer ammonium nitrate; V+N: non-concentrated vinasse plus ammonium nitrate; and CV+N: concentrated vinasse plus ammonium nitrate. Figure S7. Absolute abundance (number of reads) of soil bacterial and archaea phyla (>0.3% of the community) in sugarcane soils in the rainy season (RS). The treatments are: Control; N: inorganic fertilizer ammonium nitrate; V+N: non-concentrated vinasse plus ammonium nitrate; and CV+N: concentrated vinasse plus ammonium nitrate. The value of each bacterial group is the mean of soil samples collected from three different replicates. Means followed by the same letter in each phylum at each treatment do not differ significantly by the Tukey’s test (Significant difference: *p* ≤ 0.05; ns: Non-significant). Figure S8. Absolute abundance (number of reads) of soil bacterial and archaea phyla (>0.3% of the community) in sugarcane soils in the dry season (DS). The treatments are: Control; N: inorganic fertilizer ammonium nitrate; V+N: non-concentrated vinasse plus ammonium nitrate; and CV+N: concentrated vinasse plus ammonium nitrate. The value of each bacterial group is the mean of soil samples collected from three different replicates. Means followed by the same letter in each phylum at each treatment do not differ significantly by the Tukey’s test (Significant difference: *p* ≤ 0.05; ns: Non-significant). Figure S9. Absolute abundance (number of reads) of soil fungal phyla (>0.3% of the community) in sugarcane soils in the rainy (a) and dry season (b). The treatments are: Control; N: inorganic fertilizer ammonium nitrate; V+N: non-concentrated vinasse plus ammonium nitrate; and CV+N: concentrated vinasse plus ammonium nitrate. The value of each bacterial group is the mean of soil samples collected from three different replicates. Means followed by the same letter in each phylum at each treatment do not differ significantly by the Tukey’s test (Significant difference: *p* ≤ 0.05; ns: Non-significant). Table S1. Primers and thermocycler conditions used in gene abundance analysis by qPCR and amplicon sequence. Table S2. Alpha diversity of the ammonia-oxidizing bacterial community in treatment and timepoint comparisons. The treatments were: Control; N: inorganic N fertilizer; CV+N: concentrated vinasse plus inorganic N fertilizer; V+N: non-concentrated vinasse plus inorganic N fertilizer. Table S3. ANOSIM and PERMANOVA results generated from four different datasets: mineral and organic amendments (Bacterial, Fungal, *nirK*-Fungi, and *amoA*-AOB). Three different factors were analyzed as possible drivers of fungal microbial community structure: Treatments, days after mineral and vinasse application and interaction between both. Differences in community structure were assessed using Bray–Curtis dissimilarity. ANOSIM R scores indicate the extent of difference between two groups (e.g. treatments vs. days samples). An R value of 1 indicates that the groups share no OTUs in common. Hellinger transformation and OUT table rarefied .Table S4. F and p value of two-factor analysis of variance (ANOVA) of 16S rRNA and 18S rRNA copies numbers per gram of dry soil with day and treatment as factors. Table S5. Gene copies numbers per gram of dry soil (* 10^6^ ± standard error, g^−1^ dry soil) of total bacteria (16S rRNA) and total fungi (18S rRNA) obtained by qPCR from soil with sugarcane in different treatments in (a, b, c) rainy and (d, e, f) dry seasons. The treatments are: Control; N: mineral N fertilizer, ammonium nitrate; V+N: non-concentrated vinasse plus mineral N; CV+N: concentrated vinasse plus mineral N (n = 3).

## References

[1] Lourenço K.S., Suleiman A.K.A., Pijl A., van Veen J.A., Cantarella H., Kuramae E.E. (2018). Resilience of the resident soil microbiome to organic and inorganic amendment disturbances and to temporary bacterial invasion. Microbiome.

[2] Mattiazzo M.E., da Glória N.A. (1987). Effect of vinasse on soil acidity. Water Sci. Technol..

[3] Chen C., Zhang J., Lu M., Qin C., Chen Y., Yang L., Huang Q., Wang J., Shen Z., Shen Q. (2016). Microbial communities of an arable soil treated for 8 years with organic and inorganic fertilizers. Biol. Fertil. Soils.

[4] Franco-Andreu L., Gómez I., Parrado J., García C., Hernández T., Tejada M. (2017). Soil biology changes as a consequence of organic amendments subjected to a severe drought. Land Degrad. Dev..

[5] Sukitprapanon T.-S., Jantamenchai M., Tulaphitak D., Vityakon P. (2020). Nutrient composition of diverse organic residues and their long-term effects on available nutrients in a tropical sandy soil. Heliyon.

[6] Zhao S., Qiu S., Xu X., Ciampitti I.A., Zhang S., He P. (2019). Change in straw decomposition rate and soil microbial community composition after straw addition in different long-term fertilization soils. Appl. Soil Ecol..

[7] Suleiman A.K.A., Lourenço K.S., Clark C., Luz R.L., da Silva G.H.R., Vet L.E.M., Cantarella H., Fernandes T.V., Kuramae E.E. (2020). From toilet to agriculture: Fertilization with microalgal biomass from wastewater impacts the soil and rhizosphere active microbiomes, greenhouse gas emissions and plant growth. Resour. Conserv. Recycl..

[8] Cornwell W.K., Ackerly D.D. (2009). Community assembly and shifts in plant trait distributions across an environmental gradient in coastal California. Ecol. Monogr..

[9] Lourenço K.S., Suleiman A.K.A., Pijl A., Cantarella H., Kuramae E.E. (2020). Dynamics and resilience of soil mycobiome under multiple organic and inorganic pulse disturbances. Sci. Total Environ..

[10] Rogeri D.A., Ernani P.R., Mantovani A., Lourenço K.S. (2016). Composition of Poultry Litter in Southern Brazil. Rev. Bras. Ciência Solo.

[11] Fuess L.T., Garcia M.L., Zaiat M. (2018). Seasonal characterization of sugarcane vinasse: Assessing environmental impacts from fertirrigation and the bioenergy recovery potential through biodigestion. Sci. Total Environ..

[12] Hannula E.S., Morriën E. (2022). Will fungi solve the carbon dilemma?. Geoderma.

[13] Boer W.d., Folman L.B., Summerbell R.C., Boddy L. (2005). Living in a fungal world: Impact of fungi on soil bacterial niche development. FEMS Microbiol. Rev..

[14] Ai C., Zhang S., Zhang X., Guo D., Zhou W., Huang S. (2018). Distinct responses of soil bacterial and fungal communities to changes in fertilization regime and crop rotation. Geoderma.

[15] Banerjee S., Kirkby C.A., Schmutter D., Bissett A., Kirkegaard J.A., Richardson A.E. (2016). Network analysis reveals functional redundancy and keystone taxa amongst bacterial and fungal communities during organic matter decomposition in an arable soil. Soil Biol. Biochem..

[16] Suleiman A.K.A., Gonzatto R., Aita C., Lupatini M., Jacques R.J.S., Kuramae E.E., Antoniolli Z.I., Roesch L.F.W. (2016). Temporal variability of soil microbial communities after application of dicyandiamide-treated swine slurry and mineral fertilizers. Soil Biol. Biochem..

[17] Mutton M.A., Rossetto R., Mutton M.J.R., Cortez L.A.B. (2014). Agricultural use of stillage. Sugarcane Bioethanol—R&D for Productivity and Sustainability.

[18] CONAB (2020). Acompanhamento da Safra Brasileira de Cana-de-Açúcar: V. 7—SAFRA 2020/21 N.1—Primeiro levantamento|MAIO 2020.

[19] Lourenço K.S., Rossetto R., Vitti A.C., Montezano Z.F., Soares J.R., de Melo Sousa R., do Carmo J.B., Kuramae E.E., Cantarella H. (2019). Strategies to mitigate the nitrous oxide emissions from nitrogen fertilizer applied with organic fertilizers in sugarcane. Sci. Total Environ..

[20] Critchfield H.J. (1960). General Climatology.

[21] Staff S.S., USDA (2014). Keys to Soil Taxonomy. Soil Survey Staff.

[22] FAO (2015). World reference base for soil resources 2014, update 2015. International Soil Classification System for Naming Soils and Creating Legends for Soil Maps.

[23] Camargo O.A., Moniz A.C., Jorge J.A., Valadares J.M. (1986). Methods of soil Chemical, Physical, and Mineralogical Analysis of the Agronomic Institute in Campinas.

[24] Van Raij B., Andrade J.C., Cantarella H., Quaggio J.A. (2001). Chemical Analysis for Evaluation of Fertility of Tropical Soils.

[25] Lourenço K.S., Dimitrov M.R., Pijl A., Soares J.R., do Carmo J.B., van Veen J.A., Cantarella H., Kuramae E.E. (2018). Dominance of bacterial ammonium oxidizers and fungal denitrifiers in the complex nitrogen cycle pathways related to nitrous oxide emission. GCB Bioenergy.

[26] Hollander M.D. (2017). Nioo-Knaw/Hydra: 1.3.3.

[27] Koster J., Rahmann S. (2012). Snakemake—A scalable bioinformatics workflow engine. Bioinformatics.

[28] Bushnell B. (2015). BBMap. http://sourceforge.net/projects/bbmap/.

[29] Rognes T., Flouri T., Nichols B., Quince C., Mahé F. (2016). VSEARCH: A versatile open source tool for metagenomics. PeerJ.

[30] Bengtsson-Palme J., Ryberg M., Hartmann M., Branco S., Wang Z., Godhe A., De Wit P., Sánchez-García M., Ebersberger I., de Sousa F. (2013). Improved software detection and extraction of ITS1 and ITS2 from ribosomal ITS sequences of fungi and other eukaryotes for analysis of environmental sequencing data. Methods Ecol. Evol..

[31] Edgar R.C. (2010). Search and clustering orders of magnitude faster than BLAST. Bioinformatics.

[32] Edgar R.C., Haas B.J., Clemente J.C., Quince C., Knight R. (2011). UCHIME improves sensitivity and speed of chimera detection. Bioinformatics.

[33] McDonald D., Clemente J.C., Kuczynski J., Rideout J.R., Stombaugh J., Wendel D., Wilke A., Huse S., Hufnagle J., Meyer F. (2012). The Biological Observation Matrix (BIOM) format or: How I learned to stop worrying and love the ome-ome. GigaScience.

[34] Pruesse E., Peplies J., Glöckner F.O. (2012). SINA: Accurate high-throughput multiple sequence alignment of ribosomal RNA genes. Bioinformatics.

[35] Kõljalg U., Nilsson R.H., Abarenkov K., Tedersoo L., Taylor A.F.S., Bahram M., Bates S.T., Bruns T.D., Bengtsson-Palme J., Callaghan T.M. (2013). Towards a unified paradigm for sequence-based identification of fungi. Mol. Ecol..

[36] McMurdie P.J., Holmes S. (2013). phyloseq: An R package for reproducible interactive analysis and graphics of microbiome census data. PLoS ONE.

[37] Chao A. (1984). Nonparametric estimation of the number of classes in a population. Scand. J. Stat..

[38] Oksanen J., Blanchet F.G., Friendly M., Kindt R., Legendre P., McGlinn D., Minchin P.R., O’Hara R.B., Simpson G.L., Solymos P. (2017). Vegan: Community Ecology Package. R Package Version 2.4-4. https://cran.r-project.org/web/packages/vegan/vegan.pdf.

[39] Dray S., Dufour A.B. (2007). The ade4 package: Implementing the duality diagram for ecologists. J. Stat. Softw..

[40] Gower J.C. (1975). Generalized procrustes analysis. Psychometrika.

[41] Paliy O., Shankar V. (2016). Application of multivariate statistical techniques in microbial ecology. Mol. Ecol..

[42] Peres-Neto P.R., Jackson D.A. (2001). How well do multivariate data sets match? The advantages of a Procrustean superimposition approach over the Mantel test. Oecologia.

[43] Robert P., Escoufier Y. (1976). A unifying tool for linear multivariate statistical methods: The *RV*-coefficient. J. R. Stat. Soc..

[44] Dray S., Chessel D., Thioulouse J. (2003). Co-inertia analysis and the linking of ecological data tables. Ecology.

[45] Dhariwal A., Chong J., Habib S., King I.L., Agellon L.B., Xia J. (2017). MicrobiomeAnalyst: A web-based tool for comprehensive statistical, visual and meta-analysis of microbiome data. Nucleic Acids Res..

[46] Carmo J.B.d., Filoso S., Zotelli L.C., de Sousa Neto E.R., Pitombo L.M., Duarte-Neto P.J., Vargas V.P., Andrade C.A., Gava G.J.C., Rossetto R. (2013). Infield greenhouse gas emissions from sugarcane soils in Brazil: Effects from synthetic and organic fertilizer application and crop trash accumulation. GCB Bioenergy.

[47] Suleiman A.K.A., Lourenço K.S., Pitombo L.M., Mendes L.W., Roesch L.F.W., Pijl A., Carmo J.B., Cantarella H., Kuramae E.E. (2018). Recycling organic residues in agriculture impacts soil-borne microbial community structure, function and N_2_O emissions. Sci. Total Environ..

[48] Pitombo L.M., do Carmo J.B., de Hollander M., Rossetto R., López M.V., Cantarella H., Kuramae E.E. (2016). Exploring soil microbial 16S rRNA sequence data to increase carbon yield and nitrogen efficiency of a bioenergy crop. GCB Bioenergy.

[49] Güsewell S., Gessner M.O. (2009). N:P ratios influence litter decomposition and colonization by fungi and bacteria in microcosms. Funct. Ecol..

[50] Attermeyer K., Hornick T., Kayler Z.E., Bahr A., Zwirnmann E., Grossart H.P., Premke K. (2014). Enhanced bacterial decomposition with increasing addition of autochthonous to allochthonous carbon without any effect on bacterial community composition. Biogeosciences.

[51] Bardgett R.D., McAlister E. (1999). The measurement of soil fungal:bacterial biomass ratios as an indicator of ecosystem self-regulation in temperate meadow grasslands. Biol. Fertil. Soils.

[52] Gessner M.O., Gulis V., Kuehn K.A., Chauvet E., Suberkropp K., Kubicek C.P., Druzhinina I.S. (2007). Fungal decomposers of plant litter in aquatic ecosystems. Environmental and Microbial Relationships.

[53] Taylor C.R., Hardiman E.M., Ahmad M., Sainsbury P.D., Norris P.R., Bugg T.D. (2012). Isolation of bacterial strains able to metabolize lignin from screening of environmental samples. J. Appl. Microbiol..

[54] Kämpfer P., Dworkin M., Falkow S., Rosenberg E., Schleifer K.-H., Stackebrandt E. (2006). The Family *Streptomycetaceae*, Part I: Taxonomy. The Prokaryotes: Volume 3: Archaea. Bacteria: Firmicutes, Actinomycetes.

[55] Cassman N.A., Lourenço K.S., do Carmo J.B., Cantarella H., Kuramae E.E. (2018). Genome-resolved metagenomics of sugarcane vinasse bacteria. Biotechnol. Biofuels.

[56] Cipriano M.A.P., Suleiman A.K.A., da Silveira A.P.D., do Carmo J.B., Kuramae E.E. (2019). Bacterial community composition and diversity of two different forms of an organic residue of bioenergy crop. PeerJ.

[57] Coenye T., Rosenberg E., DeLong E.F., Lory S., Stackebrandt E., Thompson F. (2014). The Family *Burkholderiaceae*. The Prokaryotes: Alphaproteobacteria and Betaproteobacteria.

[58] Compant S., Nowak J., Coenye T., Clément C., Ait Barka E. (2008). Diversity and occurrence of *Burkholderia* spp. in the natural environment. FEMS Microbiol. Rev..

[59] Muangthong A., Youpensuk S., Rerkasem B. (2015). Isolation and characterisation of endophytic nitrogen fixing bacteria in sugarcane. Trop. Life Sci. Res..

[60] Leite M.F.A., Dimitrov M.R., Freitas-Iório R.P., de Hollander M., Cipriano M.A.P., Andrade S.A.L., da Silveira A.P.D., Kuramae E.E. (2021). Rearranging the sugarcane holobiont via plant growth-promoting bacteria and nitrogen input. Sci. Total Environ..

[61] Middelhoven W.J. (2005). *Trichosporon wieringae* sp.nov., an anamorphic basidiomycetous yeast from soil, and assimilation of some phenolic compounds, polysaccharides and other non-conventional carbon sources by saprophytic *Trichosporon species*. Antonie Leeuwenhoek.

[62] Middelhoven W.J., Scorzetti G., Fell J.W. (2001). *Trichosporon porosum* comb. nov., an anamorphic basidiomycetous yeast inhabiting soil, related to theloubieri/laibachiigroup of species that assimilate hemicelluloses and phenolic compounds. FEMS Yeast Res..

[63] Freitas P.V., da Silva D.R., Beluomini M.A., da Silva J.L., Stradiotto N.R. (2018). Determination of phenolic acids in sugarcane vinasse by HPLC with pulse amperometry. J. Anal. Methods Chem..

[64] Parnaudeau V., Condom N., Oliver R., Cazevieille P., Recous S. (2008). Vinasse organic matter quality and mineralization potential, as influenced by raw material, fermentation and concentration processes. Bioresour. Technol..

[65] Fonseca Á., Boekhout T., Fell J.W., Kurtzman C.P., Fell J.W., Boekhout T. (2011). Chapter 138—*Cryptococcus Vuillemin* (1901). The Yeasts.

[66] Chang C.-F., Lee C.-F., Liu S.-M. (2008). *Cryptococcus keelungensis* sp. nov., an anamorphic basidiomycetous yeast isolated from the sea-surface microlayer of the north-east coast of Taiwan. Int. J. Syst. Evol. Microbiol..

[67] Liu X.Z., Wang Q.M., Göker M., Groenewald M., Kachalkin A.V., Lumbsch H.T., Millanes A.M., Wedin M., Yurkov A.M., Boekhout T. (2015). Towards an integrated phylogenetic classification of the *Tremellomycetes*. Stud. Mycol..

[68] Zhang S., Wang Y., Sun L., Qiu C., Ding Y., Gu H., Wang L., Wang Z., Ding Z. (2020). Organic mulching positively regulates the soil microbial communities and ecosystem functions in tea plantation. BMC Microbiol..

