# Supplementary material for: Carbon and Nutrients from Organic Residues Modulate the Dynamics of Prokaryotic and Fungal Communities
Source: Microorganisms. 2023 Dec 1;11(12):2905. doi: 10.3390/microorganisms11122905 (PMC10745876; doi:10.3390/microorganisms11122905)
Supplement: Supplementary file 1 [file microorganisms-11-02905-s001.zip › microorganisms-2723663-supplementary.pdf]

Carbon and Nutrients from Organic Residues Modulate the Dynamics of Prokaryotic and Fungal Communities

Késia Silva Lourenço, Heitor Cantarella and Eiko Eurya Kuramae

Supplementary Material

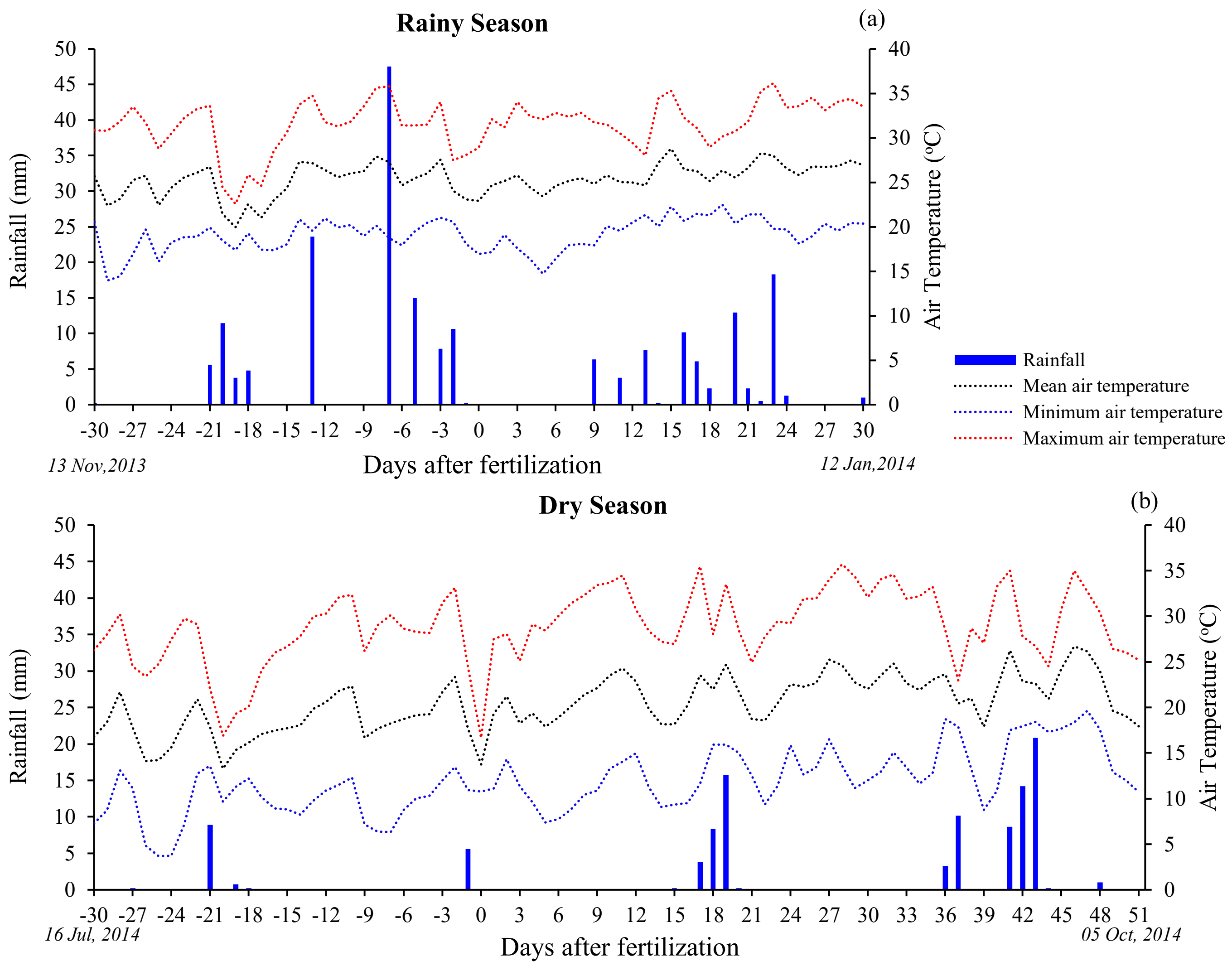

Figure S1. Rainfall (mm) and air temperature (°C) measured in the (a) rainy and (b) dry seasons.

The mean temperatures during the 30-day and 50-day experiment were 25.9 °C and 21.7 °C in the RS and DS experiments, respectively (Figure S1). The lowest air temperature were 14.7°C and 7.4 °C and the highest 36.2 °C and 35.7 °C in the RS and DS experiments, respectively. However, in the month before fertilization the minimum temperature was 14 and 3.7°C in the RS and DS experiments, respectively. The precipitation one month before the sampling days were 131 and 16 mm in the RS and DS, respectively. The period between rain events in the DS was usually higher than 15 days. On the contrary in the RS, the highest period without rain events was 10 days, it was right after fertilization. However, in the 7 days before fertilization rained 81 mm.

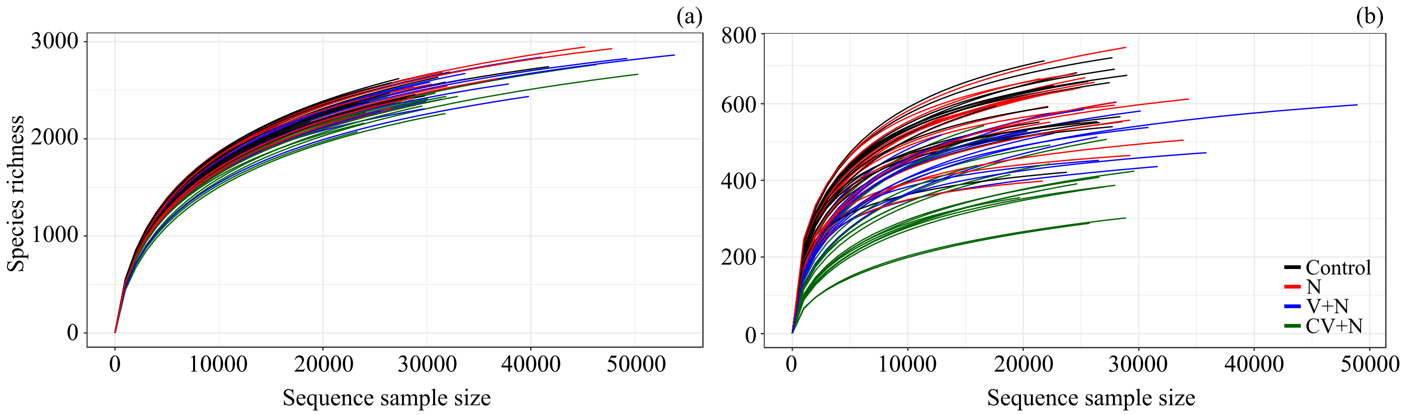

Figure S2. Rarefaction curves from non-rarefied data from the bacterial plus archaea (a) and fungi (b) communities in each treatment. The treatments were: Control; N: inorganic fertilizer ammonium nitrate; V+N: non-concentrated vinasse plus ammonium nitrate; and CV+N: concentrated vinasse plus ammonium nitrate.

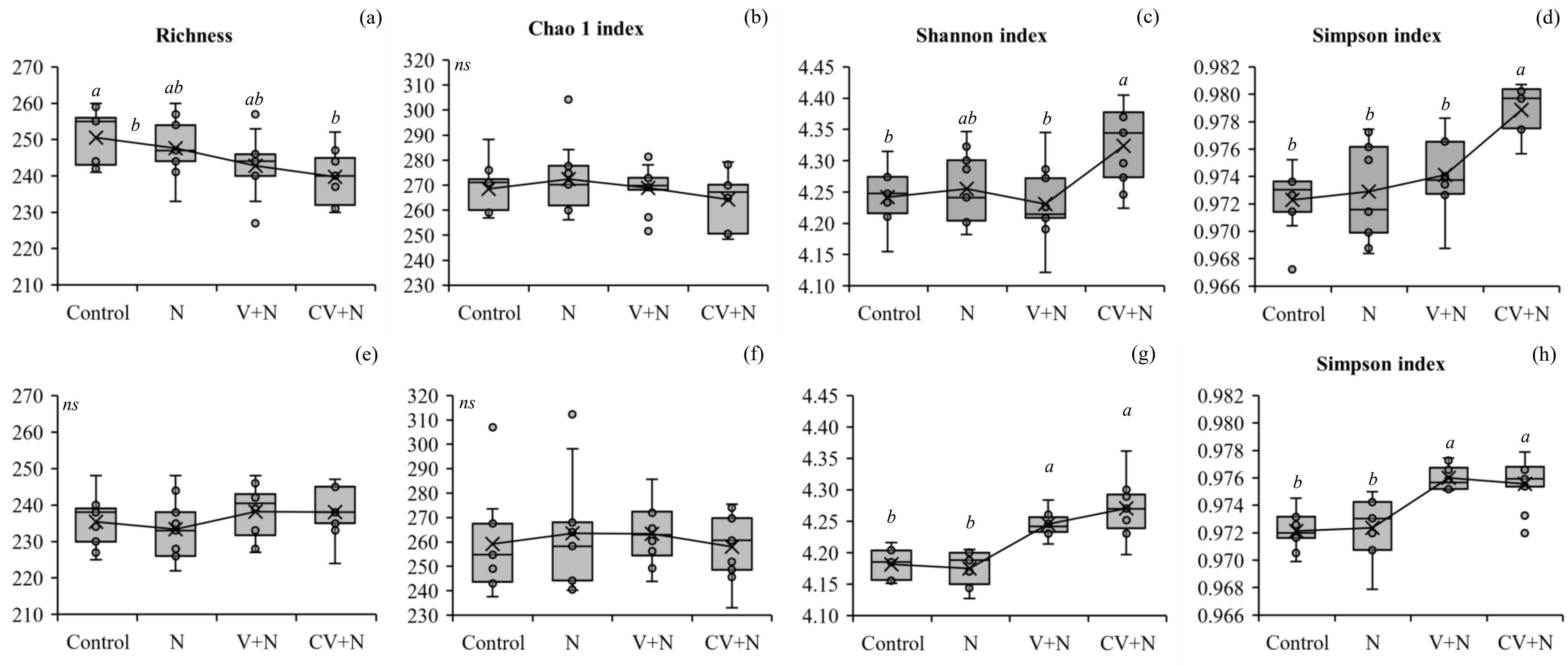

Figure S3. Alpha diversity of the bacterial and archaea families in different treatments in the rainy (a, b, c, d) and dry season (e, f, g, h), without timepoint comparisons. The treatments were: Control; N: inorganic N fertilizer; V+N: non-concentrated vinasse plus inorganic N fertilizer; and CV+N: concentrated vinasse plus inorganic N fertilizer. Means followed by the same letter at each treatment do not differ significantly by the Tukey's test (Significant difference: $p \leq 0.05$; ns: Non-significant).

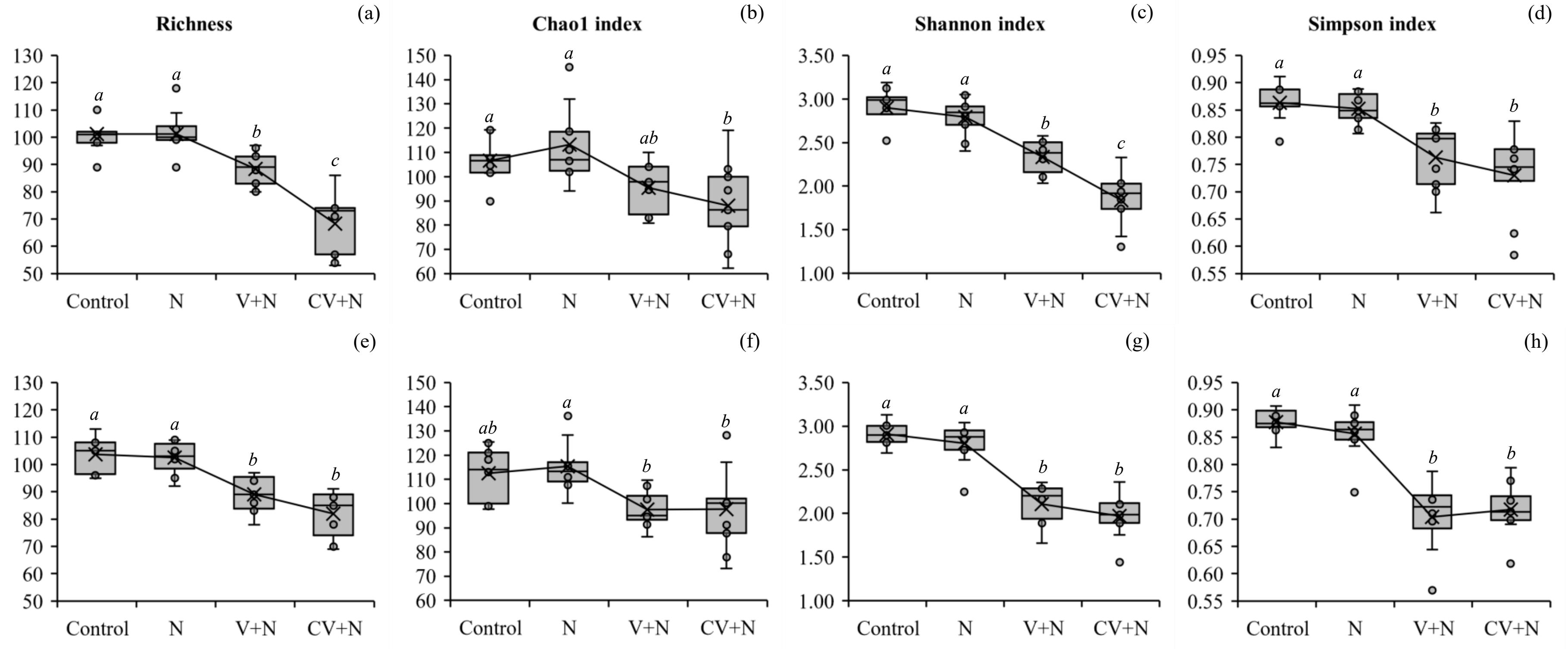

Figure S4. Alpha diversity of the total fungal families in different treatments in the rainy and dry season (a, b, c, d), without timepoint comparisons. The treatments were: Control; N: inorganic N fertilizer; V+N: non-concentrated vinasse plus inorganic N fertilizer; and CV+N: concentrated vinasse plus inorganic N fertilizer. Means followed by the same letter at each treatment do not differ significantly by the Tukey's test (Significant difference: $p \leq 0.05$; ns: Non-significant).

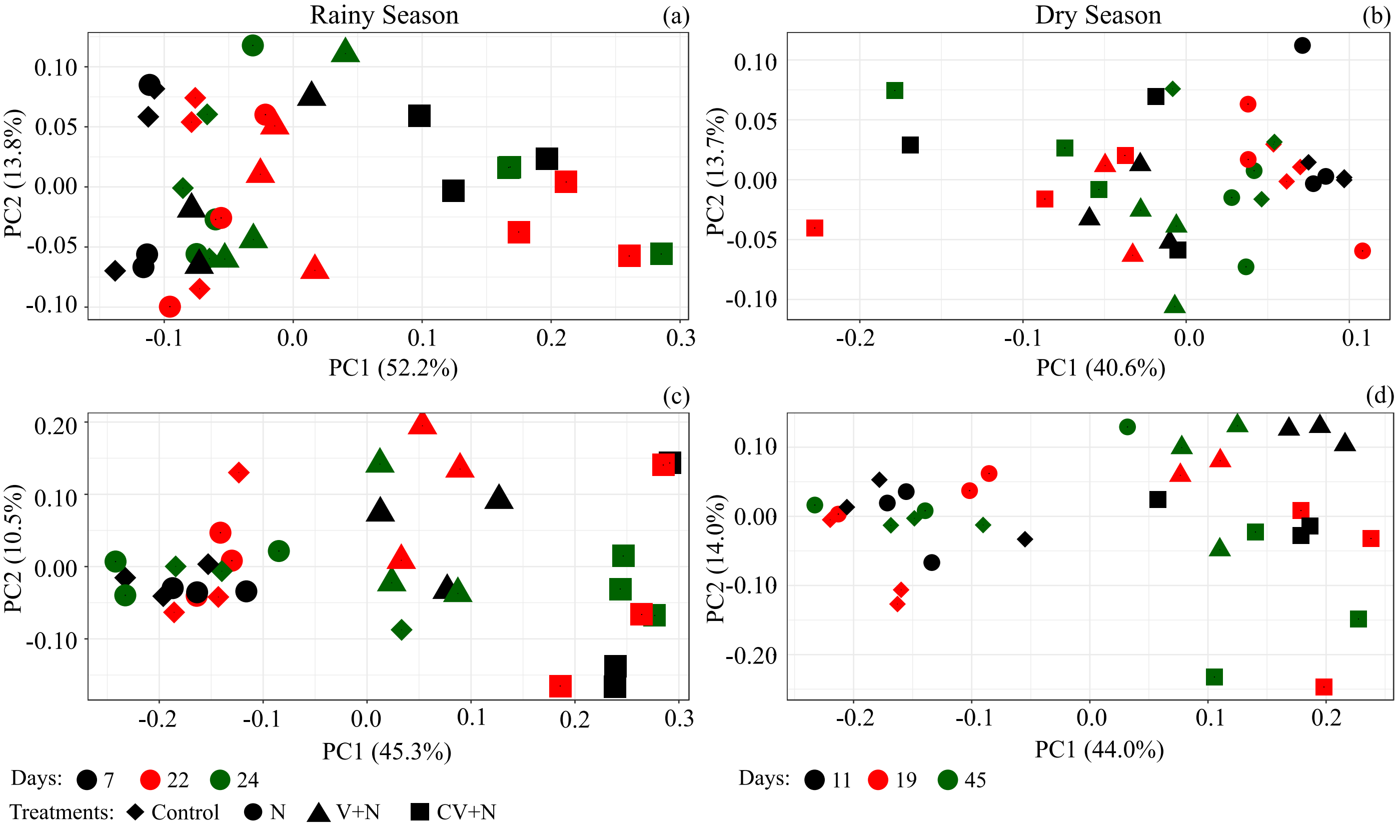

Figure S5. Temporal changes in the soil bacterial and archaea (a, b), and total fungal (c, d) communities in the rainy (a, c) and dry (b, d) season as depicted by Bray-Curtis dissimilarity (which accounts for changes in the relative abundance of families). Principal coordinate analysis (PCoA) of soils cultivated with sugarcane was performed with three time points. To illustrate the differences between days in each treatment, each time point was showed with different colour. The treatments were as follows: Control; N: inorganic fertilizer ammonium nitrate; V+N: non-concentrated vinasse plus ammonium nitrate; and CV+N: concentrated vinasse plus ammonium nitrate. Each point represents an individual sample, with colors indicating days. The positions of the points are the average for the jackknife replicates. OUT table rarefied.

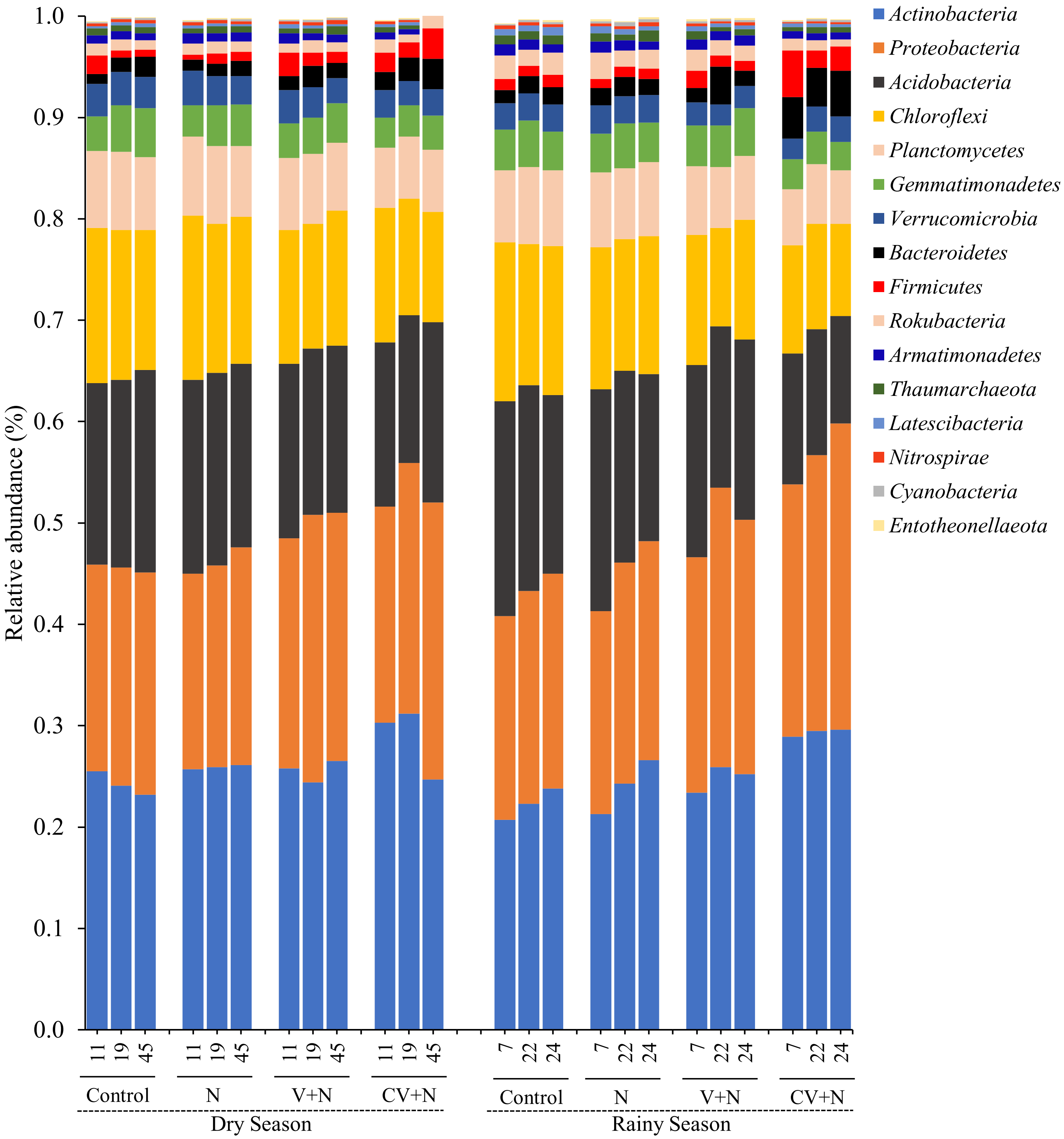

Figure S6. Sequence abundance phylum of Bacteria and Archaea Domains (>0.002) using normalized values between 0 and 1 (%) for Control; N: inorganic fertilizer ammonium nitrate; V+N: non-concentrated vinasse plus ammonium nitrate; and CV+N: concentrated vinasse plus ammonium nitrate.

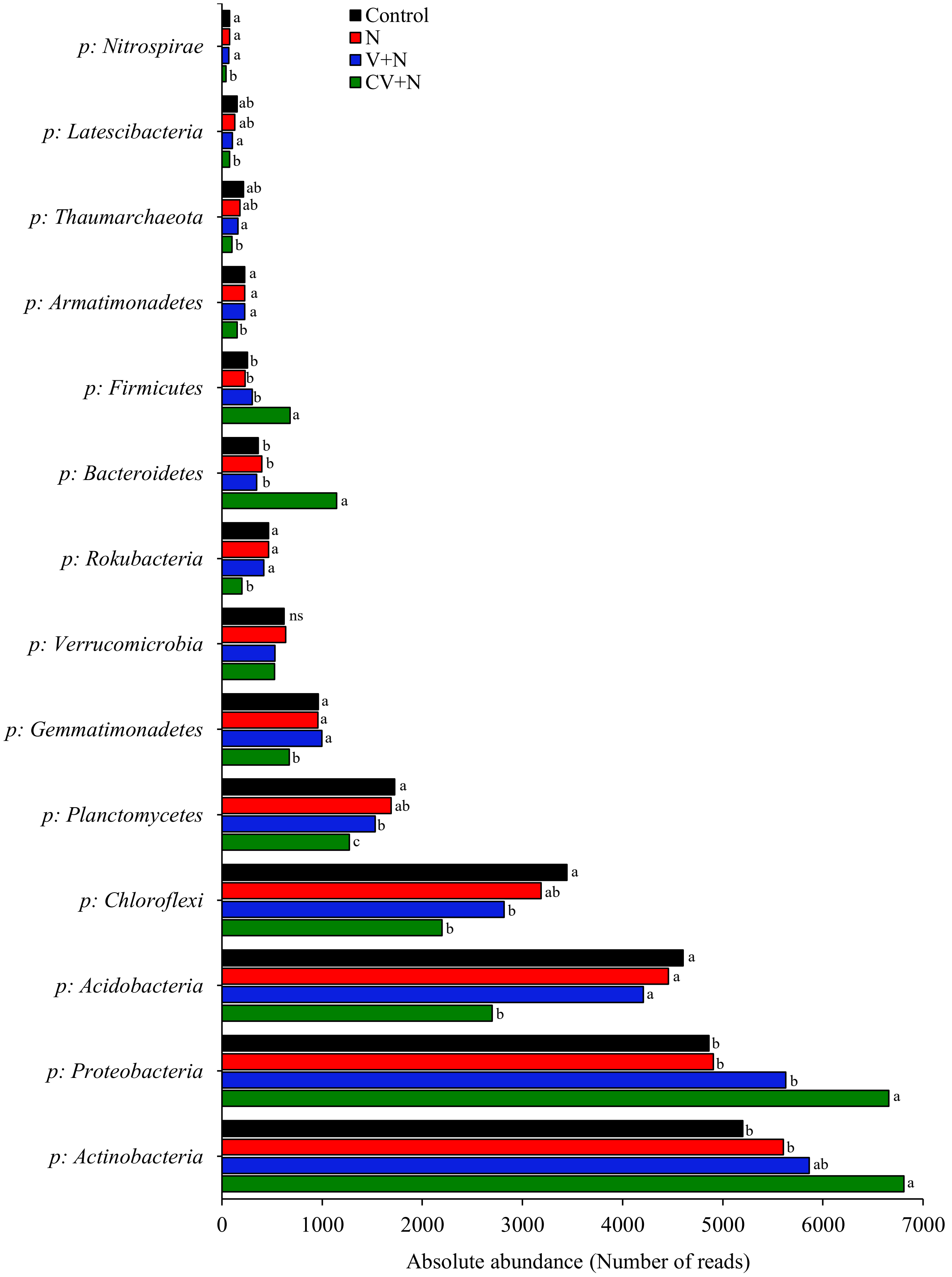

Figure S7. Absolute abundance (number of reads) of soil bacterial and archaea phyla (>0.3% of the community) in sugarcane soils in the rainy season (RS). The treatments are: Control; N: inorganic fertilizer ammonium nitrate; V+N: non-concentrated vinasse plus ammonium nitrate; and CV+N: concentrated vinasse plus ammonium nitrate. The value of each bacterial group is the mean of soil samples collected from three different replicates. Means followed by the same letter in each phylum at each treatment do not differ significantly by the Tukey's test (Significant difference: p≤ 0.05; ns: Non-significant).

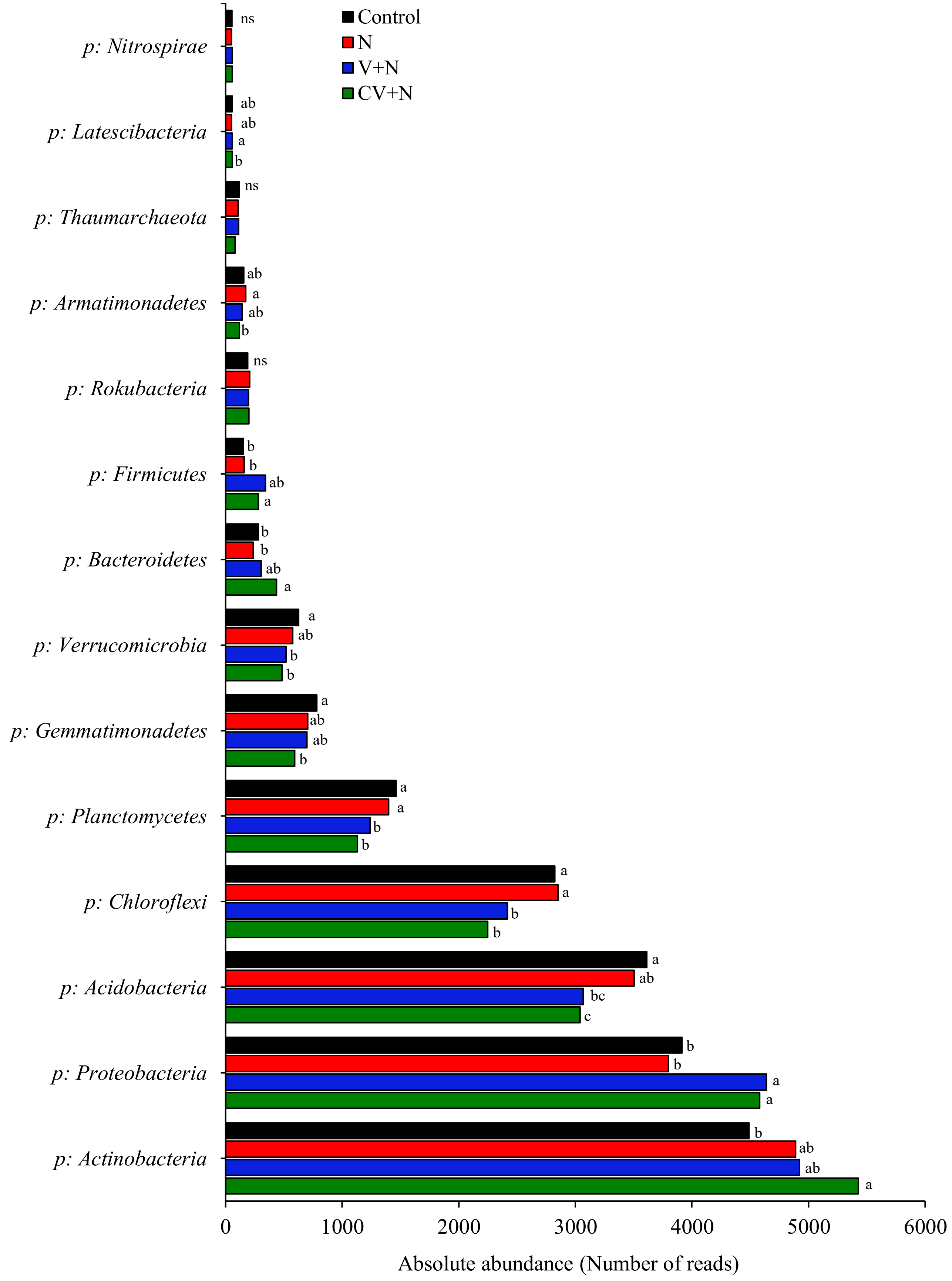

Figure S8. Absolute abundance (number of reads) of soil bacterial and archaea phyla (>0.3% of the community) in sugarcane soils in the dry season (DS). The treatments are: Control; N: inorganic fertilizer ammonium nitrate; V+N: non-concentrated vinasse plus ammonium nitrate; and CV+N: concentrated vinasse plus ammonium nitrate. The value of each bacterial group is the mean of soil samples collected from three different replicates. Means followed by the same letter in each phylum at each treatment do not differ significantly by the Tukey's test (Significant difference: $p \leq 0.05$; ns: Non-significant).

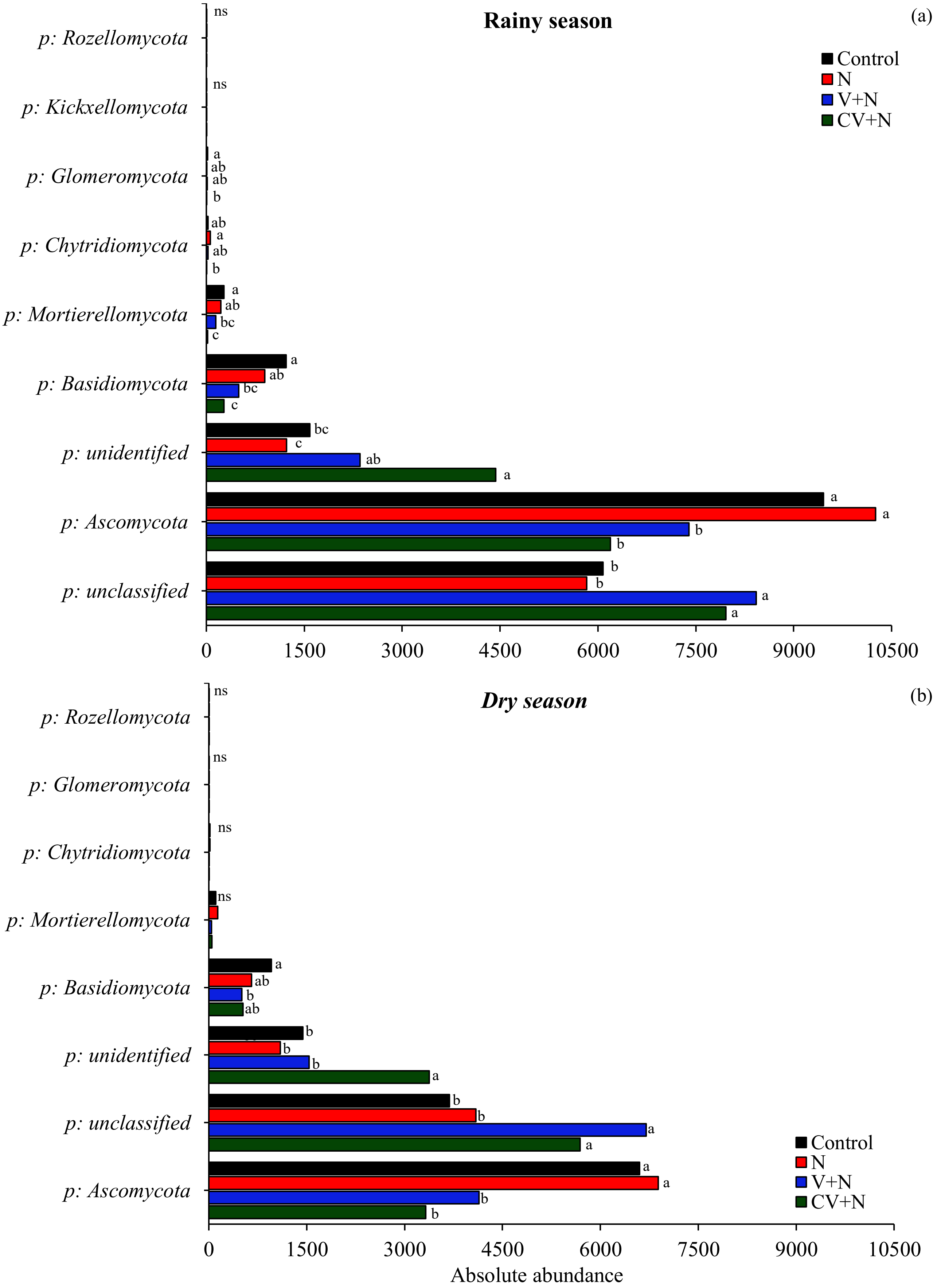

Figure S9. Absolute abundance (number of reads) of soil fungal phyla (>0.3% of the community) in sugarcane soils in the rainy (a) and dry season (b). The treatments are: Control; N: inorganic fertilizer ammonium nitrate; V+N: non-concentrated vinasse plus ammonium nitrate; and CV+N: concentrated vinasse plus ammonium nitrate. The value of each bacterial group is the mean of soil samples collected from three different replicates. Means followed by the same letter in each phylum at each treatment do not differ significantly by the Tukey's test (Significant difference: $p \leq 0.05$; ns: Non-significant).

Table S1. Primers and thermocycler conditions used in gene abundance analysis by qPCR and amplicon sequence.

Target gene	Primers	Primer Sequence	Amplification size (bp)	Reaction	Cycling conditions	
**qPCR**				**12 µL of reaction**		
16S rRNA	Eub338<br>Eub518	5'-ACTCCTACGGGAGGCAGCAG-3'<br>5'-ATTACCGCGGCTGCTGG-3'	200	6 µL of Sybrgreen iQ™ SYBR® Green Supermix (Bio-Rad), 0.125 µL of each primer (10 pmol), 0.30 µL of BSA and 4 µL of DNA (5 ng).	95°C-3 min.; 40x 95°C-30s, 59°C-35s, 72°C-20s	(Fierer et al., 2005)
18S rRNA	FF390<br>FFR1	5'-CGATAACGAACGAGACCT-3'<br>5'-AICCATTCAATCGGTAIT-3'	390	6 µL of Sybrgreen iQ™ SYBR® Green Supermix (Bio-Rad), 0.250 µL of each primer (10 pmol), 0.30 µL of BSA and 4 µL of DNA (5 ng).	95°C-3 min.; 40x 95°C-30s, 52°C-45 s, 72°C-50 s	Vainio and Hantula (2000)
PCR amplification				**8 µL of reaction**		
16S rRNA	515FP1-CS1<br>515FP2-CS1<br>515FP3-CS1<br>515FP4-CS1<br>806RP1-CS2<br>806RP2-CS2<br>806RP3-CS2<br>806RP4-CS2	5'-ACACTGACGACATGGTTCTACAGTGCCAGCMGCCGCGGTAA-3'<br>5'-ACACTGACGACATGGTTCTACATGTGCCAGCMGCCGCGGTAA-3'<br>5'-ACACTGACGACATGGTTCTACAACGTGCCAGCMGCCGCGGTAA-3'<br>5'-ACACTGACGACATGGTTCTACACTAGTGCCAGCMGCCGCGGTAA-3'<br>5'-TACGGTAGCAGAGACTTGGTCTGGACTACHVGGGTWTCTAAT-3'<br>5'-TACGGTAGCAGAGACTTGGTCTTGGACTACHVGGGTWTCTAAT-3'<br>5'-TACGGTAGCAGAGACTTGGTCTACGGACTACHVGGGTWTCTAAT-3'<br>5'-TACGGTAGCAGAGACTTGGTCTCTAGGACTACHVGGGTWTCTAAT-3'	291	0.80 µL of Roche PCR Buffer 10X with 18 mM $MgCl_2$, 0.40 µL of Roche DMSO, 0.16 µL of KAPA dNTP mix (10 mM), 0.03 µL of Taq 5U- µL (Roche_FastStart High Fi), 5.51 µL of H_2O, 0.05 µL of each primer (10 pmol) and 1 µL of DNA (1/50 Dilution /Conc. Used).	94°C-2 min.; 33x 94°C-30s, 58°C-30s, 72ºC-30s; and 72ºC-7 min.	(Caporaso et al., 2012)
				**8 µL of reaction**		
ITS	ITS1FP1-CS1<br>ITS1FP2-CS1<br>ITS1FP3-CS1<br>ITS1FP4-CS1<br>ITS2P1-CS2<br>ITS2P2-CS2<br>ITS2P3-CS2<br>ITS2P4-CS2	5'-ACACTGACGACATGGTTCTACACTTGGTCATTTAGAGGAAGTAA-3'<br>5'-ACACTGACGACATGGTTCTACATCTTGGTCATTTAGAGGAAGTAA-3'<br>5'-ACACTGACGACATGGTTCTACAACCTTGGTCATTTAGAGGAAGTAA-3'<br>5'-ACACTGACGACATGGTTCTACACTACTTGGTCATTTAGAGGAAGTAA-3'<br>5'-TACGGTAGCAGAGACTTGGTCTGCTGCGTTCTTCATCGATGC-3'<br>5'-TACGGTAGCAGAGACTTGGTCTTGCTGCGTTCTTCATCGATGC-3'<br>5'-TACGGTAGCAGAGACTTGGTCTACGCTGCGTTCTTCATCGATGC-3'<br>5'-TACGGTAGCAGAGACTTGGTCTCTAGCTGCGTTCTTCATCGATGC-3'		0.8 µL of Qiagen_PCR 10X Buffer with15 mM $MgCl_2$, 0.40 µL of Roche DMSO, 0.16 µL of dNTP mix 10 mM_FroggaBio, 0.03 µL of Taq 5U-ul Qiagen HotStarTaq, 5.54 µL of H_2O, 0.03 µL of each primer (10 pmol) and 1 µL of DNA (1/50 Dilution /Conc. Used).	96°C-15min; 33x 96°C-30s, 52°C-30s, 72°C-60 s; and 72°C-10 min;	

Table S2. Alpha diversity of the ammonia-oxidizing bacterial community in treatment and timepoint comparisons. The treatments were: Control; N: inorganic N fertilizer; CV+N: concentrated vinasse plus inorganic N fertilizer; V+N: non-concentrated vinasse plus inorganic N fertilizer.

ANOVA test [a]	Richness	Chao1	Shannon	Simpson
**Rainy Season**				
	Bacterial community			
Treatment	**	*ns*	***	***
Day	*ns*	*	**	**
Treatment x Day	*ns*	*ns*	*ns*	*ns*
	Fungal community			
Treatment	***	***	***	***
Day	*ns*	*ns*	*ns*	*ns*
Treatment x Day	*ns*	*ns*	*ns*	*ns*
**Dry Season**				
	Bacterial community			
Treatment	*ns*	*ns*	***	***
Day	*	*ns*	*ns*	*ns*
Treatment x Day	*ns*	*ns*	*ns*	*ns*
	Fungal community			
Treatment	***	***	***	***
Day	*ns*	*ns*	*ns*	*ns*
Treatment x Day	*ns*	*ns*	*	*

[a] Symbols in the caption refer to overall ANOVA results for the given experiment. Significant difference: * p≤ 0.10, ** p≤ 0.05, *** p≤ 0.01 and ns: Non-Significant.

Table S3. ANOSIM and PERMANOVA results generated from four different datasets: mineral and organic amendments (Bacterial, Fungal, *nirK*-Fungi, and *amoA*-AOB). Three different factors were analyzed as possible drivers of fungal microbial community structure: Treatments, days after mineral and vinasse application and interaction between both. Differences in community structure were assessed using Bray–Curtis dissimilarity. ANOSIM R scores indicate the extent of difference between two groups (*e.g.* treatments vs. days samples). An R value of 1 indicates that the groups share no OTUs in common. Hellinger transformation and OUT table rarefied

**Factor**	**Analysis**	***Df***	**F. Model**	**R**	**R^2**	**p-value**
**Rainy season**						
			Bacteria			
Treatment	ANOSIM	-	-	0.53	-	P = 0.00
	PERMANOVA	3	9.57	-	0.45	P = 0.00
Day	ANOSIM	-	-	0.01	-	P = 0.30
	PERMANOVA	1	3.79	-	0.06	P = 0.01
Treatment*Day	PERMANOVA	3	1.00	-	0.05	P = 0.41
			Fungi			
Treatment	ANOSIM	-	-	0.75	-	P = 0.00
	PERMANOVA	3	11.02	-	0.51	P = 0.00
Day	ANOSIM	-	-	-0.05	-	P = 0.96
	PERMANOVA	1	1.09	-	0.02	P = 0.29
Treatment*Day	PERMANOVA	3	1.05	-	0.04	P = 0.36
**Dry season**						
			Bacteria			
Treatment	ANOSIM	-	-	0.39	-	P = 0.00
	PERMANOVA	3	4.88	-	0.30	P = 0.00
Day	ANOSIM	-	-	0.07	-	P = 0.04
	PERMANOVA	1	3.20	-	0.07	P = 0.00
Treatment*Day	PERMANOVA	3	1.15	-	0.07	P = 0.245
			Fungi			
Treatment	ANOSIM	-	-	0.66	-	P = 0.00
	PERMANOVA	3	7.16	-	0.41	P = 0.00
Day	ANOSIM	-	-	-0.04	-	P = 0.83
	PERMANOVA	1	1.19	-	0.23	P = 0.24
Treatment*Day	PERMANOVA	6	0.79.90	-	0.05	P = 0.54

Table S4. F and p value of two-factor analysis of variance (ANOVA) of 16S rRNA and 18S rRNA copies numbers per gram of dry soil with day and treatment as factors.

	Rainy Season		Dry Season	
ANOVA	F value	p	F value	p
16S rRNA				
Day	**11.44**	**0.0003**	**10.19**	**0.0005**
Treatments	1.01	0.4070	1.07	0.3799
Day*Treatments	0.79	0.5871	0.32	0.9225
18S rRNA				
Day	0.89	0.4231	1.08	0.3552
Treatments	**32.77**	**0.0000**	**6.50**	**0.0022**
Day*Treatments	1.00	0.4487	0.86	0.5385

Table S5. Gene copies numbers per gram of dry soil (* 10^6 ± standard error, g^{-1} dry soil) of total bacteria (16S rRNA) and total fungi (18S rRNA) obtained by qPCR from soil with sugarcane in different treatments in (a, b, c) rainy and (d, e, f) dry seasons. The treatments are: Control; N: mineral N fertilizer, ammonium nitrate; V+N: non-concentrated vinasse plus mineral N; CV+N: concentrated vinasse plus mineral N (n = 3).

	Treatments				
**Days***	**Control**	**N**	**V+N**	**CV+N**	*Average*
16S rRNA					
			Rainy season		
7	1376.6 ±473.7	1458.5 ±269.8	1990.8 ±266.1	1967.9 ±421.2	*1698.5 ±357.7B*
22	5017.4 ±988.3	5215.8 ±730.1	3709.7 ±1679.2	7445.0 ±2153.9	*5347.0 ±1387.9A*
24	3785.8 ±1156.4	3623.1 ±711.5	5395.8 ±1648.5	4927.1 ±1023.3	*4433.0 ±1134.9A*
Average	*3393.3 ±872.8*	*3432.5 ±570.5*	*3698.8 ±1198.0*	*4780.0 ±1199.5*	
			Dry season		
11	5784.16 ±706.4	7068.77 ±819.4	4131.76 ±827.1	8907.69 ±3207.6	*6473 ±1390.1A*
19	5843.09 ±1413.2	6772.77 ±3396.2	5474.96 ±1785.1	7581.68 ±2816.0	*6418 ±2352.6A*
45	968.18 ±219.0	1114.58 ±177.3	1834.10 ±593.2	2104.55 ±118.0	*1505 ±276.9B*
Average	*4198.5 ±779.5*	*4985.4 ±1464.3*	*3813.6 ±1068.4*	*6198.0 ±2047.2*	
18S rRNA					
			Rainy season		
7	5.9 ±1.6	7.6 ±0.7	12.4 ±2.8	98.4 ±25.5	*31.1 ±7.6*
22	13.2 ±2.4	14.8 ±1.3	8.4 ±2.4	107.4 ±23.9	*35.9 ±7.5*
24	12.2 ±4.8	11.8 ±1.5	10.7 ±4.8	64.0 ±20.6	*24.7 ±7.9*
Average	*10.4 ±3.0b*	*11.4 ±1.2b*	*10.5 ±3.3b*	*89.9 ±23.3a*	
			Dry season		
11	8.7 ±2.3	10.3 ±3.4	24.4 ±9.0	104.4 ±77.1	*37 ±23.0*
19	10.6 ±1.7	11.5 ±6.1	31.0 ±7.8	184.9 ±88.2	*60 ±26.0*
45	6.5 ±2.2	9.4 ±1.2	26.0 ±5.9	55.3 ±11.5	*24 ±5.2*
Average	*8.6 ±2.1b*	*10.4 ±3.5b*	*27.2 ±7.6b*	*114.9 ±58.9a*	

* Means followed by same lowercase letters in the rows and uppercase letters in the columns do not differ significantly (Tukey $p \leq 0.05$).

References

Caporaso, J.G., Lauber, C.L., Walters, W.A., Berg-Lyons, D., Huntley, J., Fierer, N., Owens, S.M., Betley, J., Fraser, L., Bauer, M., et al., 2012. Ultra-high-throughput microbial community analysis on the Illumina HiSeq and MiSeq platforms. The Isme Journal 6, 1621-1624. https://doi.org/10.1038/ismej.2012.8

Fierer, N., Jackson, J.A., Vilgalys, R., Jackson, R.B., 2005. Assessment of soil microbial community structure by use of taxon-specific quantitative PCR assays. Applied and Environmental Microbiology 71, 4117-4120. https://doi.org/10.1128/aem.71.7.4117-4120.2005

Vainio, E.J., Hantula, J., 2000. Direct analysis of wood-inhabiting fungi using denaturing gradient gel electrophoresis of amplified ribosomal DNA. Mycological Research 104, 927-936. https://doi.org/10.1017/S0953756200002471
